# Efficacy and safety of berberine on the components of metabolic syndrome: a systematic review and meta-analysis of randomized placebo-controlled trials

**DOI:** 10.3389/fphar.2025.1572197

**Published:** 2025-07-16

**Authors:** Dangzhen Liu, Haiyan Zhao, Yiwen Zhang, Jingqing Hu, Hong Xu

**Affiliations:** ^1^ School of Basic Medical Sciences, Chengdu University of Traditional Chinese Medicine, Chengdu, China; ^2^ School of Third Clinical Medical, Nanjing University of Traditional Chinese Medicine, Nanjing, China; ^3^ Institute of Basic Theory for Chinese Medicine, China Academy of Chinese Medical Sciences, Beijing, China; ^4^ School of Interdisciplinary Medicine, Tianjin University of Traditional Chinese Medicine, Tianjin, China; ^5^ State Key Laboratory of Dampness Syndrome of Chinese Medicine, The Second Affiliated Hospital of Guangzhou University of Chinese Medicine, Guangzhou, China; ^6^ XIN-Huangpu Joint Innovation Institute of Chinese Medicine, Guangzhou, China

**Keywords:** metabolic syndrome, berberine, hyperglycemia, dyslipidemia, hypertension, waist circumference (WC)

## Abstract

**Background:**

Metabolic syndrome (MetS) is a prevalent metabolic disease that significantly increases the risk of type 2 diabetes, cardiovascular diseases, and overall mortality. Current medications have limited effects on the various components of MetS. Berberine has demonstrated unique comprehensive therapeutic benefits for MetS, but its efficacy remains uncertain.

**Objective:**

To comprehensively evaluate the efficacy and safety of berberine on MetS indicators.

**Methods:**

Our study provides a comprehensive evaluation of the efficacy and safety of berberine for MetS components through systematic review and meta-analysis, from the aspects of study characteristics, risk of bias, meta-analysis, sensitivity analysis, meta-regression, and publication bias.

**Results:**

The results indicate that berberine significantly reduces triglycerides (TG) (WMD: −0.367 mmol/L; 95% CI: −0.560 to −0.175; *p* < 0.001), fasting plasma glucose (FPG) (WMD: −0.515 mmol/L; 95% CI: −0.847 to −0.183; *p* = 0.002), and waist circumference (WC) (WMD: −3.270 cm; 95% CI: −4.818 to −1.722; *p* < 0.001) among the components of MetS, but has no significant effect on high-density lipoprotein cholesterol (HDL-C), systolic blood pressure (SBP), or diastolic blood pressure (DBP). Additionally, berberine also improves the secondary indicators of low-density lipoprotein cholesterol (LDL-C) (WMD: −0.495 mmol/L; 95% CI: −0.714 to −0.276; *p* < 0.001), total cholesterol (TC) (WMD: −0.451 mmol/L; 95% CI: −0.631 to −0.271; *p* < 0.001), body mass index (BMI) (WMD: −0.435 kg/m^2^; 95% CI: −0.856 to −0.013; *p* = 0.043), and 2-h oral glucose tolerance (2hOGTT) (WMD: −1.606 mmol/L; 95% CI: −1.891 to −1.321; *p* < 0.001). Meta-regression and subgroup analyses indicate that short-term treatment (≤90 days) is more effective for HDL-C and LDL-C than long-term treatment. Regarding safety, no significant difference was observed between berberine and placebo.

**Conclusion:**

Berberine significantly improves glucose and lipid metabolism and has notable effects on components of MetS, including TG, FPG, and WC, with a favorable safety profile. It may serve as a beneficial supplement. Meanwhile, more high-quality, rigorously designed randomized controlled trials are needed in the future to provide stronger evidence.

**Systematic review registration:**

https://www.crd.york.ac.uk/PROSPERO/view/CRD42024588614.

## 1 Introduction

Metabolic syndrome (MetS) is a multifactorial metabolic disorder. It is defined by the presence of at least three of the following five risk factors: abdominal obesity, hypertension, hyperglycemia, high triglycerides (TG), and low high-density lipoprotein cholesterol (HDL-C) (Alberti et al., 2005; Alberti et al., 2009). It is estimated that one-quarter of the global population is affected by MetS, with projections suggesting this figure could rise to approximately 53% by 2035 (Saklayen, 2018; Belhayara et al., 2020; Collaborators, 2020). MetS increases the risk of cardiovascular events fivefold and the likelihood of developing type 2 diabetes (T2DM) twofold, while also significantly raising the risk of other chronic diseases and overall mortality (Wu et al., 2010; Sangouni et al., 2023). The underlying mechanisms of MetS are multifactorial. Insulin resistance is considered the core feature (Belhayara et al., 2020). Visceral fat accumulation, inflammation, oxidative stress, and endothelial dysfunction all contribute significantly to the disorder (Takahara and Shimomura, 2014; Sharebiani et al., 2024).

Berberine has been shown to have the potential to treat MetS. Several studies have confirmed that berberine possesses properties that ameliorate dyslipidemia, insulin resistance, and hypertension (Turner et al., 2008; Xu et al., 2014; Xu et al., 2017; Wang et al., 2018; Rondanelli et al., 2023; Yan et al., 2024). It exerts its effects through the activation of AMP-activated protein kinase, enhancing glucose uptake in peripheral tissues, promoting lipid metabolism, and influencing fasting plasma glucose (FPG) and TG levels (Turner et al., 2008; Xu et al., 2014; Wang et al., 2018). Additionally, berberine reduces reactive oxygen species production and increases antioxidant enzyme activity, thereby mitigating oxidative stress that contributes to hyperglycemia (Zhang et al., 2022; Rafiei et al., 2023). Berberine has also been shown to increase HDL-C levels, exhibiting lipid-modulating properties (Rondanelli et al., 2023). Research has demonstrated that berberine downregulates the TMAO-mediated endoplasmic reticulum stress pathway, improving vascular dysfunction and lowering blood pressure (Yan et al., 2024). In various experimental models, berberine has been shown to reduce adipose tissue mass and inhibit fat accumulation (Dong et al., 2015). A 2022 meta-analysis investigating the effects of berberine on adult cardiovascular risk factors included components of MetS (Zamani et al., 2022), but the treatment groups involved combination therapies (Guarino et al., 2017) and extracts from Berberis plants (Shidfar et al., 2011; Zilaee et al., 2014; Asemani et al., 2018; Aryaeian et al., 2020), and the safety of berberine was not evaluated. This inconsistency and lack of comprehensiveness may lead to uncertainties regarding the effects of berberine on MetS components. Moreover, there are recent randomized controlled trials (RCTs) on the effects of berberine on MetS components (Wu et al., 2022; Panigrahi and Mohanty, 2023; Ran, 2023). Therefore, there is a need to update and comprehensively synthesize these studies to clarify the efficacy and safety of purified berberine.

This study presents a systematic review and meta-analysis of RCTs comparing purified berberine versus placebo. The efficacy of berberine was evaluated using the components of MetS, with secondary outcomes including the 2-h oral glucose tolerance test (2hOGTT) (Bergman et al., 2023), total cholesterol (TC), low-density lipoprotein cholesterol (LDL-C), and body mass index (BMI), along with an assessment of its safety. Throughout this review, “efficacy on MetS” denotes improvements in the individual diagnostic components of metabolic syndrome only; reversal of the syndrome in its entirety was not evaluated.

## 2 Materials and methods

This study was conducted in accordance with the Preferred Reporting Items for Systematic Reviews and Meta-Analyses (PRISMA) statement (Page et al., 2021) and was registered in PROSPERO (registration number: CRD42024588614 https://www.crd.york.ac.uk/PROSPERO/display_record.php?RecordID=588614).

### 2.1 Search strategy

We performed a systematic search in PubMed, Web of Science, Embase, Cochrane Library, and China National Knowledge Infrastructure (from the inception of the databases to 15 November 2024, with no language restrictions) to identify all randomized controlled trials evaluating berberine treatment for MetS. MeSH terms and free text terms were used as appropriate for each database. The detailed search strategy is provided in Supplementary Material S1. Two reviewers (D.L. and H.Z.) independently selected studies based on the PICOS framework (Participants, Intervention, Comparison, Outcomes, and Study Design): (1) Participants: Adults who meet at least one of the diagnostic criteria for MetS. (2) Intervention: Berberine. (3) Comparison: Placebo. (4) Outcomes: FPG, TG, HDL-C, LDL-C, systolic blood pressure (SBP), diastolic blood pressure (DBP), waist circumference (WC), TC, BMI, 2hOGTT, any adverse effects. (5) Study Design: RCTs.

### 2.2 Eligibility criteria

Inclusion criteria: (1) The experimental group receives berberine as the sole intervention for MetS indicators; (2) The study design is a randomized controlled trial; (3) The study is a placebo-controlled design; (4) When multiple studies involve the same population, the most comprehensive and rigorous study is selected.

Exclusion criteria: (1) Studies with incomplete data; (2) There are combined treatments targeting MetS indicators; (3) Review, letters to the editor, and conference abstracts.

### 2.3 Data extraction and risk of bias assessment

According to the Cochrane Reviewer’s Handbook (Higgins et al., 2011), the data extraction was carried out independently by D.L. and H.Z., and any discrepancies were resolved through consultation with Y.Z. If information was incomplete or unclear, the corresponding authors were contacted as necessary. The following data were extracted: (1) study characteristics (first author, publication date, country, study population, total sample size, study design, presence of dietary and exercise guidance); (2) participant characteristics (mean age, gender); (3) intervention and comparison data (intervention method, dosage, intervention duration, any adverse effects); (4) outcome measures (mean values and standard deviations [SD] for TG, HDL-C, LDL-C, BMI, TC, FPG, WC, SBP, DBP, and 2hOGTT before and after treatment).

The quality of the included studies was assessed using the Cochrane Collaboration’s Risk of Bias Tool version 2 (RoB 2) for randomized controlled trials (Sterne et al., 2019). The RoB 2 tool evaluates the following domains: randomization processes, deviations from intended interventions, missing outcome data, measurements of the outcomes, selection of the reported results, and overall bias. If methodological errors were identified in any domain, it was rated as “high risk”; if no errors were found, it was rated as “low risk”; and if insufficient data were available for evaluation, a “some concerns” rating was applied. The processes of study selection and data extraction were conducted independently by D.L. and H.Z., and the results were cross-checked.

### 2.4 Data synthesis and analysis

All statistical analyses were conducted using STATA 17.0. Meta-analysis is performed using baseline and post-treatment means and SDs from both the intervention and placebo groups. For consistency, when the treatment of a trail study is divided into two phases with a washout period between them, such as the study by Wang et al. (2016b), only the pre- and post-treatment data from the first treatment phase is included in our analysis. Safety statistical analysis was performed using Python 3.12.

Since the measurement units are consistent across studies, the effect size is expressed as the weighted mean difference (WMD), with the 95% confidence interval (CI) reported. Heterogeneity is assessed using the I^2^ statistic, with I^2^ > 50% indicating bigger heterogeneity (Higgins et al., 2003). A random-effects model is used for I^2^ > 50%, and a fixed-effects model is applied for I^2^ ≤ 50%. Subgroup analysis is conducted when at least six studies were included (Fe et al., 2011), based on the population using berberine (dyslipidemia or other diseases), dosage (≤0.9 g/day, 1 g/day, ≥1.5 g/day), and intervention duration (84 days, 90 days, ≥112 days), to identify potential factors influencing the effectiveness of berberine treatment and sources of heterogeneity. Meta-regression is used to assess the potential impact of age (20–39 years or 40–59 years), sex (male-predominant (>52%), female-predominant (number of male <48%, the 48%–52% male participation range is absent across all included studies), berberine dose (g/day) and duration on outcome measures, also helping to identify sources of heterogeneity. Sensitivity analysis is performed to assess the influence of individual studies on the overall pooled effect size. Funnel plots, Egger’s test, and Begg-Mazumdar correlation tests are employed to detect publication bias. If bias is detected, a “trim and fill” analysis is used to correct it. In all analyses, a *p*-value of less than 0.05 is considered statistically significant.

## 3 Results

### 3.1 Study selection

We searched five databases, identifying 1 903 studies. After removing 397 duplicate articles, we excluded 1 506 studies based on title and abstract not meeting inclusion/exclusion criteria (irrelevant titles and abstracts [n = 97], animal and cell studies [n = 523], improper comparison [n = 611], non-randomized controlled trials [n = 57], comments, letters, and conference abstracts [n = 3], reviews [n = 176]). We then assessed 39 potentially relevant studies for eligibility.

**FIGURE 1:**
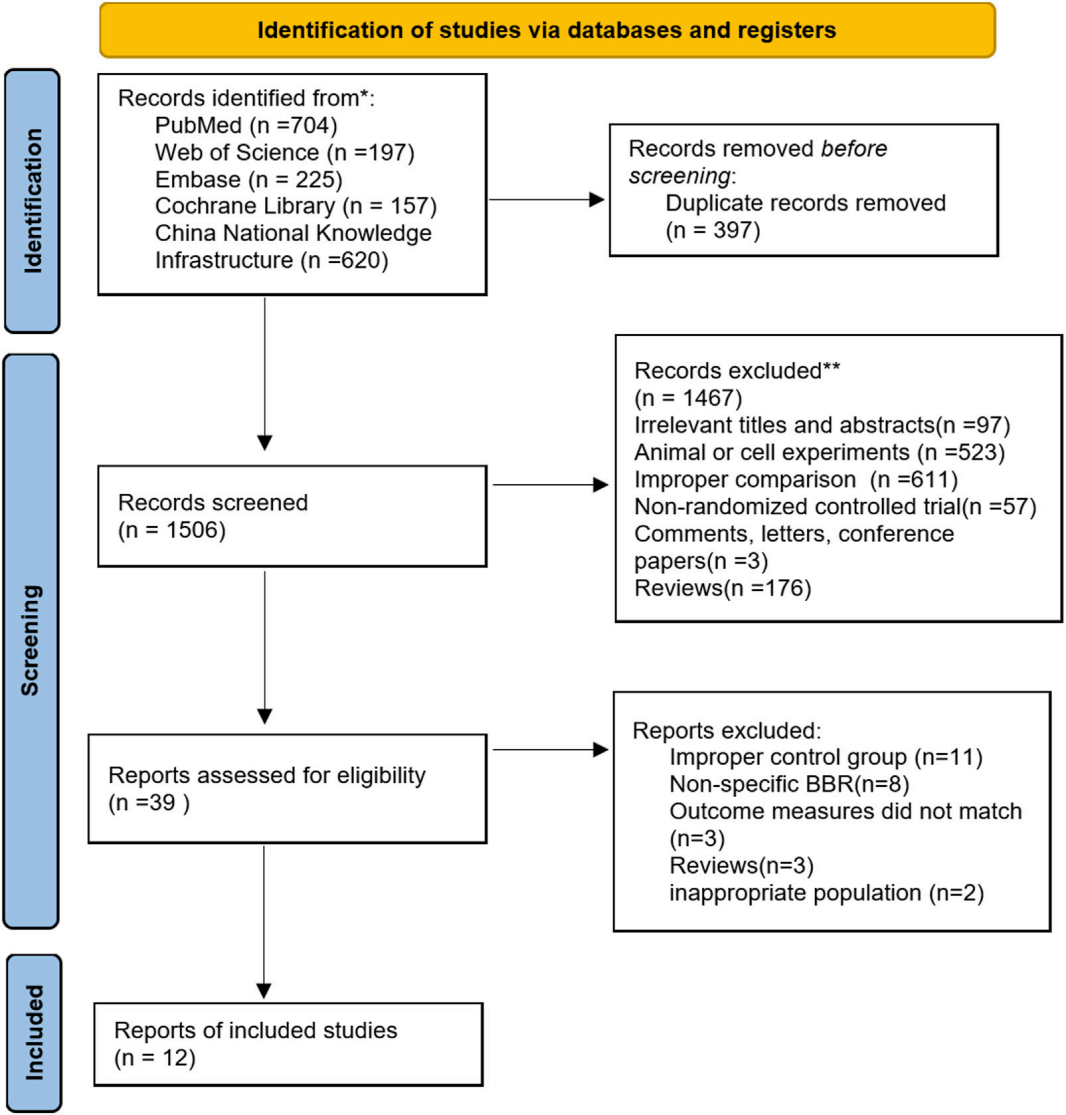
PRISMA flow diagram of selecting studies.

Of these, 27 studies were excluded for the following reasons: (1) improper control group; (2) non-specific berberine; (3) outcome measures did not match; (4) reviews; (5) inappropriate population. Notably, specific berberine refers to berberine with a purity greater than 95% and not combined with other substances. Eight studies involving non-specific berberine, such as Berberis plant or root extracts (Shidfar et al., 2011; Zilaee et al., 2014; Iloon Kashkooli et al., 2015; Asemani et al., 2018; Aryaeian et al., 2020; Rostami et al., 2023) and berberine-phospholipid complexes (Rondanelli et al., 2023; Cesarone et al., 2024), were excluded because they were non-purified berberine or other complexes of berberine. Additionally, when two studies reported data from the same participant group, the one with less detailed information was excluded, to avoid duplication in the meta-analysis. For this reason, one study was excluded as having an inappropriate population (Gu et al., 2010). Ultimately, 12 randomized placebo-controlled trials (RCTs) on berberine were included. The detailed procedures of the study search and screening are presented in Figure 1.

### 3.2 Study characteristics and drug safety

This meta-analysis is based on a total of twelve randomized, placebo-controlled trials published between 2004 and 2023, conducted in China, Mexico, and India, with the majority taking place in China. These trials involved populations with dyslipidemia (Kong et al., 2004; Wang et al., 2016a; Zhao et al., 2021b; Wu et al., 2022), T2DM-associated dyslipidemia (Zhang et al., 2008), hyperglycemia (Ming et al., 2021), drug-induced impaired fasting glucose (IFG) (Pu et al., 2021), prediabetes (Panigrahi and Mohanty, 2023), polycystic ovary syndrome (PCOS) with infertility (An et al., 2014), high HDL-C syndrome (Ran, 2023), metabolic syndrome (Perez-Rubio et al., 2013), and HIV-infected individuals with metabolic syndrome (Ruiz-Herrera et al., 2023). The total sample size across the studies is 889 participants, and all studies are parallel placebo-controlled trials.

About dietary and exercise guidance, five trials provided such guidance to all participants during the study period (Zhang et al., 2008; An et al., 2014; Ming et al., 2021; Wu et al., 2022; Ruiz-Herrera et al., 2023), one provided dietary guidance only (Perez-Rubio et al., 2013), two trials did not alter participants’ diet or lifestyle habits (Kong et al., 2004; Panigrahi and Mohanty, 2023), and the rest studies did not report such information. Regarding age information, two studies reported an average participant age under 40 years, with the mean ages being 37.50 years (Perez-Rubio et al., 2013) and 28.45 years (An et al., 2014), while two studies did not report the average participant age (Kong et al., 2004; Zhang et al., 2008). The remaining studies had participant ages ranging from 40 to 60 years. Regarding gender information, one recruited only female participants (An et al., 2014), one recruited only male participants (Zhao et al., 2021a), and three did not report participant gender characteristics (Kong et al., 2004; Wu et al., 2022; Wang et al., 2016b). The remaining studies recruited both male and female participants. In all the studies, berberine was administered in tablet or capsule form, with dosages ranging from 300 to 1,500 mg/day and intervention durations between 84 and 140 days.

Of the 12 eligible trials, 9 assessed TG (Kong et al., 2004; Zhang et al., 2008; Perez-Rubio et al., 2013; An et al., 2014; Pu et al., 2021; Zhao et al., 2021a; Wu et al., 2022; Ran, 2023; Wang et al., 2016b), 9 assessed HDL-C (Kong et al., 2004; Zhang et al., 2008; Perez-Rubio et al., 2013; An et al., 2014; Ming et al., 2021; Pu et al., 2021; Zhao et al., 2021a; Wu et al., 2022; Wang et al., 2016b), 8 assessed LDL-C (Kong et al., 2004; Zhang et al., 2008; An et al., 2014; Ming et al., 2021; Pu et al., 2021; Zhao et al., 2021a; Wu et al., 2022; Wang et al., 2016b), 8 assessed BMI (Zhang et al., 2008; Perez-Rubio et al., 2013; An et al., 2014; Ming et al., 2021; Pu et al., 2021; Zhao et al., 2021b; Ruiz-Herrera et al., 2023; Wang et al., 2016b), 7 assessed TC (Zhang et al., 2008; An et al., 2014; Ming et al., 2021; Pu et al., 2021; Zhao et al., 2021a; Wu et al., 2022; Wang et al., 2016b), 6 assessed FPG (Zhang et al., 2008; An et al., 2014; Ming et al., 2021; Pu et al., 2021; Panigrahi and Mohanty, 2023; Wang et al., 2016b), 4 assessed WC (Perez-Rubio et al., 2013; An et al., 2014; Ming et al., 2021; Pu et al., 2021), 3 assessed SBP and DBP (Zhang et al., 2008; Perez-Rubio et al., 2013; Zhao et al., 2021a), and 3 assessed 2hOGTT (Zhang et al., 2008; Ming et al., 2021; Panigrahi and Mohanty, 2023). The characteristics of the included studies are presented in Table 1.

**TABLE 1:** Characteristics of included studies.

Study (first author’s name andyear)	Country/po-pulartion	Number of participants (T/C)	Study design	Dietary and exercise recommendation (D/E/N/NR) *	Mean age	Gender (M/F)	Inter-vention Type	Dosage (mg)	Dura-tion (day)	Outcomes
Kong et al. (2004)	China/Dyslipidemia	43 (32/11)	parallel	N	56.95	M/F (23/20)	NR	2*500 mg daily	90	TG, HDL-C, LDL-C
Zhang et al. (2008)	China/T2DM and Dyslipidemia	110 (58/52)	parallel	D&E	NR	M/F (61/49)	Tablets	2*500 mg daily	90	SBP, DBP, TG, FPG, TC, HDL-C, LDL-C, BMI, 2hOGTT
Pérez-Rubio et al. (2013)	Mexico/MetS	24 (12/12)	parallel	D	37.50	M/F (10/14)	NR	3*500 mg daily	90	SBP, DBP, TG, WC, HDL-C, BMI
An et al. (2014)	China/PCOS with Infertility	87 (44/43)	parallel	D&E	28.45	F (87)	Tablets	3*500 mg daily	90	TG, FPG, WC, TC, HDL-C, LDL-C, BMI
Wang et al. (2016a)	China/Dyslipid-emia	89 (44/45)	parallel	D&E	56	M/F (41/48)	Capsules	3*300 mg daily	90	TG, FPG, TC, HDL-C, LDL-C,BMI
Ming et al. (2021)	China/hyperglycemia	148 (49/99)	parallel	D&E	53.01	M/F (77/71)	Tablets	2^a^500 mg daily	112	FPG, WC, TC, HDL-C, LDL-C, BMI, 2hOGTT
Pu et al. (2021)	China/IFG with olanzapine for at least 9 months	110 (58/52)	parallel	NR	43.83	M/F (79/31)	Tablets	3^a^ (100–300) mg daily	84	TG, FPG, WC, TC, HDL-C, LDL-C, BMI
Zhao et al. (2021a)	China/Dyslipidemia	80 (40/40)	parallel	NR	42.15	M (80)	Tablets	2^a^500 mg daily	84	SBP, DBP, TG, TC, HDL-C, LDL-C, BMI
Wu et al. (2022)	China/Dyslipidemia	83 (42/41)	parallel	D&E	54.02	NR	Tablets	2^a^500 mg daily	84	TG, TC, HDL-C, LDL-C
Panigrahi and Mohanty. (2023)	India/prediabetes	34 (17/17)	parallel	N	44.15	M/F (19/15)	Capsules	3^a^500 mg daily	84	FPG,2hOGTT
Ruiz-Herrera et al. (2023)	Mexico/MetS with HIV infection	36 (19/17)	parallel	D&E	46.5	M/F (22/14)	Capsules	3^a^500 mg daily	140	BMI
Ran (2023)	China/HypoHDL-emia	45 (20/25)	parallel	NR	49.46	M/F (28/17)	Tablets	2^a^500 mg daily	84	TG

^a^ Dietary and exercise recommendation: D = dietary advice, E = exercise advice, N = no intervention, NR not reported.

Abbreviation: T2DM, type 2 diabetes; MetS, metabolic syndrome; PCOS, polycystic ovary syndrome; IFG, impaired fasting glucose; HIV, human immunodeficiency virus; HypoHDL-emia, low high-density lipoproteinemia; T, number of people in treatment group; T, number of people in control group; M, number of male; F, number of female; mg, milligram.

In the 12 studies, 10 RCTs reported adverse events or safety outcomes related to berberine or placebo, while the remaining two studies (Kong et al., 2004; Wu et al., 2022) did not provide such information. Details of reported adverse events are presented in Table 2. The most reported adverse events were hypoglycemia, nausea, constipation, and mild abdominal discomfort; however, the overall incidence of adverse events and side effects was generally low. Pu et al.'s study conducted in 2021 (Pu et al., 2021) reported withdrawals due to mild to moderate adverse events. This may be related to the participants’ use of the antipsychotic drug olanzapine for at least 9 months, which increased their sensitivity to minor adverse reactions (Haro et al., 2006).

**TABLE 2:** Adverse Reactions and safety indicators.

Study (First author’s name andyear)	Adverse events	Safety indicators
Zhang et al. (2008)	Mild to moderate constipation (T, 5 cases; C, 1 case). There is no statistical significance between two groups	liver function and kidney function were normal, and ALT,AST, GGT were reduced
Pérez-Rubio et al. (2013)	PNR	NR
An et al. (2014)	Nausea (T, 9 cases; C, 4 cases), Vomiting (T, 4 cases; C, 1 case); Diarrhoea (T, 5 cases; C, 1 case), Abdominal discomfort (T, 7 cases; C, 2 cases). All of the above are mild to moderate adverse events and no statistical significance	NR
Wang et al. (2016a)	Short-term headache (1 case, lasting 1 day); Transient abdominal distension (2 cases, one of whom was in the washout period). No serious adverse effect reported	liver function and kidney function were normal
Ming et al. (2021)	Severe adverse events (T, 4 cases; C, 3 cases, no statistical significance), there is no statistical significance between two groups. Hypoglycemia event (T, 9 cases; C, 14 cases), fecal abnormalities (T, 3cases; C, 9cases), abdominal discomfort (T, 5 cases; C,7 cases), dental and oral disorders (T,0 case; C, 1 case), abnormal ECG/cardiac dysfunction (T, 3 cases; C, 2 cases), dizziness (T, 0 case; C, 1 case), upper respiratory tract infection (T, 0 case; C, 1 case), changes in body weight (T,0 case; C, 1 case); hemorrhoids (T, 0case; C, 1 case), others (T, 1 case; C, 0 case)	Blood routine/biochemistry/Urinalysis (T,1 case; C,7 cases)
Pu et al. (2021)	The most common adverse reactions were nausea (3 cases), mild stomach pain (4 cases), and constipation (1 case). Termination of the trial due to adverse reactions (T, 3 cases; C, 9 cases)	NR
Zhao et al. (2021a)	Headache (T, 1 case; C, 1 case), nausea (T, 0 case; C, 1case), vomiting (T, 0 case; C, 1 case), diarrhea (T, 1 case; C, 1 case). No serious adverse effect reported	NR
Panigrahi and Mohanty. (2023)	Nausea (T, 3 cases and Self-limited recovery; C, 0 case)	AST, ALT, ALP, Cr were normal
Ruiz-Herrera et al. (2023)	Mild diarrhea (T, 1 case; C, 0 case). Mild abdominal pain (T, 2 cases; C, 1case)	NR
Ran (2023)	Constipation (T, 2 case; C, 0 case). Diarrhea (T, 0 case; C, 1 case)	Liver function and kidney function were normal. There is no significant difference in ALT, AST, Cr between the two groups

Abbreviation: T, treatment group; C, control group; ALT, glutamic-pyruvic transaminase; AST, aspartate aminotransferase; GGT, γ-glutamyl transferase; ALP, alkaline phosphatase; Cr, serum creatinine; SE, serum electrolytes; PNR, participants reported no abnormalities; NR, not reported.

We performed quantitative synthesis of adverse event outcomes, with the rationale for data exclusions detailed in Supplementary Material 2. We performed quantitative synthesis of adverse event outcomes, focusing on absolute risk (AR) (Supplementary Figure S3.1) and relative risk (RR) (Supplementary Figure S3.2) estimates for categories with sufficient data. Our analysis focused on absolute risk (Supplementary Figure S3.1) and relative risk (Supplementary Figure S3.2) estimates for categories with sufficient data. Specifically, we included ‘fecal abnormalities’, ‘ any gastrointestinal adverse event’, and ‘any adverse event’ as these categories were reported in at least six studies. The results demonstrated pooled relative risks of 1.07 (95% CI: 0.15–6.76; p = 0.746) for any adverse event, 1.44 (95% CI: 0.85–2.42; p = 0.172) for any gastrointestinal adverse event, and 1.52 (95% CI: 0.67–3.44; p = 0.314) for stool abnormalities, all showing no statistical significance. To evaluate the robustness of findings, we conducted leave-one-out sensitivity analyses (Supplementary Figure S3.3) and subgroup analyses (Supplementary Figure S3.4) stratified by study quality, with all results remaining stable under sensitivity testing.”

### 3.3 Risk of bias assessment

Two studies among the 12 RCTs are considered high-quality due to low risk of bias in all domains (Zhang et al., 2008; Panigrahi and Mohanty, 2023). Two studies are deemed low quality. The study by Pu et al. is assessed as high risk (Pu et al., 2021) due to multiple participant dropouts and incomplete outcome data resulted from adverse reactions in both the experimental and control groups. Ran’s study (Ran, 2023) is rated as high risk, because it is a master’s thesis that lacked detailed descriptions of randomization methods and blinding procedures, and there are no related journal articles or research plans published. The remaining studies are classified as “some concerns”.

**FIGURE 2:**
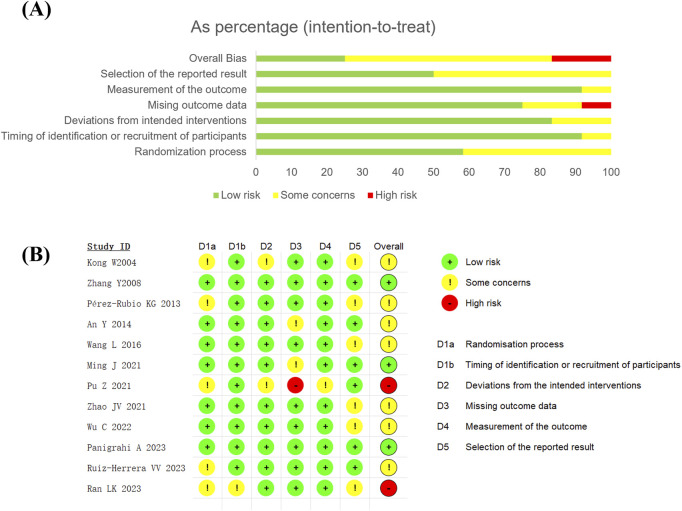
Summary plot of Cochrane Collabration’s risk of bias tool 2 of **(A)** Risk of bias summary and **(B)** Risk of bias diagram.

Of the 12 studies, one is open-label (Pu et al., 2021), and the remaining studies are double-blind. Six studies (Zhang et al., 2008; An et al., 2014; Ming et al., 2021; Pu et al., 2021; Zhao et al., 2021a; Wu et al., 2022) used computer-generated randomization methods, one study (Perez-Rubio et al., 2013) used random number tables, and the remaining studies did not report randomization methods in detail. Regarding allocation concealment, five studies (Zhang et al., 2008; An et al., 2014; Ming et al., 2021; Zhao et al., 2021a; Wu et al., 2022) employed central randomization, one study (Wang et al., 2016b) used a numbered container allocation scheme, and the remaining studies did not report allocation concealment information. The results of the quality assessment for all included studies are shown in Figure 2.

### 3.4 Meta-analysis

**FIGURE 3:**
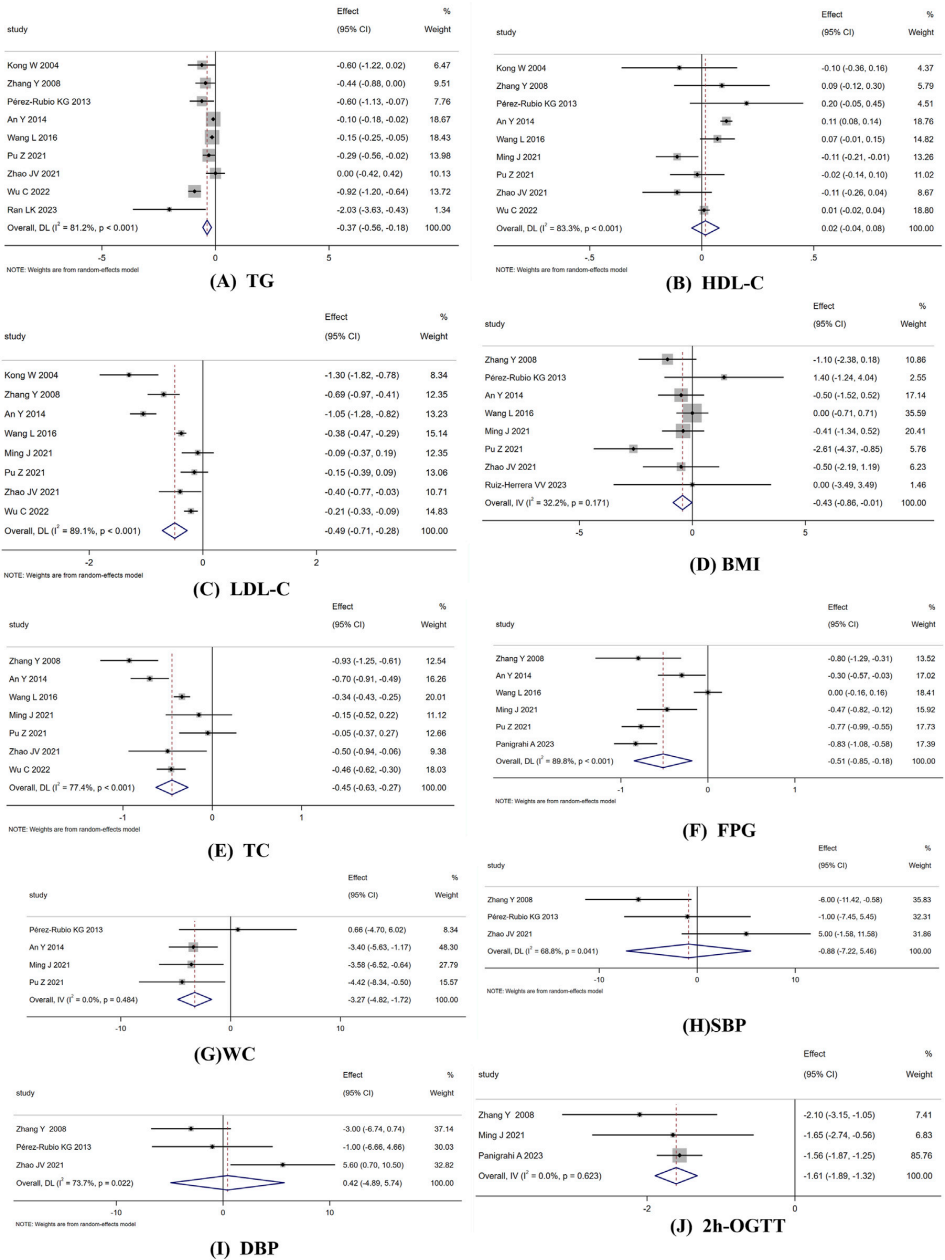
Forest plot reporting WMD and 95% CI for the efforts of berberine intake on **(A)** TG **(B)** HDL-C; **(C)** LDL-C **(D)** BMI; **(E)** TC **(F)** FPG; **(G)** WC **(H)** SBP; **(I)** DBP; **(J)** 2h-OGTT.

Nine RCTs (total sample size = 666 participants) reported on TG. The random-effects model revealed a significant reduction in TG levels with berberine intervention (WMD: −0.367 mmol/L; 95% CI: −0.560 to −0.175; *p* < 0.001), with substantial heterogeneity (I^2^ = 81.2%, *p* < 0.001) (Figure 3A). Subgroup analysis revealed that a dose of ≤1 g/day was more effective than 1.5 g/day in reducing TG. Heterogeneity decreased when stratified by dose (Table 3).

**TABLE 3:** Subgroup analyses of berberine intake on MetS parameters.

Metabolic Parameter	Subgroup	Number of studies	WMD (95%CI)	Weight (%)	*p* within group	I² (%)	*p* heterogeneity
TC (mmol/L)							
	Overall	9	-0.367(-0.560, -0.175)	100	<0.001	81.2	<0.001
	Population						
	Dyslipidemia	5	-0.513(-0.977, -0.050)	50.08	0.030	88.0	<0.001
	Other diseases	4	-0.264(-0.475, -0.052)	49.92	0.014	54.9	0.084
	Dosage (g/day)						
	<0.9	2	-0.166(-0.258, -0.074)	32.42	<0.001	0.0	0.340
	1	5	-0.596(-1.052, -0.140)	26.43	0.010	75.8	0.002
	1.5	2	-0.278(-0.747, -0.191)	41.16	0.246	69.6	0.070
	Duration (day)						
	84	4	-0.551(-1.082, -0.020)	39.16	0.042	85.0	<0.001
	90	5	-0.185(-0.307, -0.063)	60.84	0.003	47.7	0.105
HDL-C (mmol/L)							
	Overall	9	-0.016(-0.045, 0.078)	100	0.600	83.3	<0.001
	Population						
	Dyslipidemia	4	0.008(-0.050, 0.067)	46.67	0.777	43.1	0.153
	Other diseases	5	0.038(-0.08, 0.155)	53.33	0.530	82.6	<0.001
	Dosage (g/day)						
	<0.9	2	0.038(-0.047, 0.122)	25.85	0.384	33.1	0.221
	1	5	-0.04(-0.118, 0.037)	50.89	0.309	55.1	0.063
	1.5	2	0.111(0.084, 0.138)	23.26	<0.001	0.0	0.488
	Duration (day)						
	84	5	0.04(-0.050, 0.043)	38.50	0.878	17.4	0.298
	90	3	0.105(0.080, 0.129)	60.84	<0.001	0.0	0.421
	≥112	1	-0.11(-0.205, -0.015)	13.26	0.023	0.0	<0.001
LDL-C (mmol/L)							
	Overall	8	-0.495(-0.714, -0.276)	100	<0.001	89.1	<0.001
	Population						
	Dyslipidemia	4	-0.448(-0.681, -0.214)	49.01	<0.001	83.8	<0.001
	Other diseases	4	-0.497(-0.964, -0.030)	50.99	0.037	92.4	<0.001
	Dosage (g/day)						
	<0.9	2	-0.495(-0.714, -0.279)	28.20	<0.001	67.2	0.081
	1	5	-0.485(-0.802, -0.169)	58.57	0.003	84.8	<0.001
	1.5	1	-1.050(-1.282, -0.818)	13.23	<0.001	0.0	<0.001
	Duration(day)						
	84	3	-0.214(-0.318, -0.110)	38.59	<0.001	0.00	0.541
	90	4	-0.819(-1.240, -0.398)	49.05	<0.001	92.4	<0.001
	≥112	1	-0.090(-0.372, 0.192)	12.35	0.531	0.00	<0.001
BMI (Kg/m²)							
	Overall	8	-0.435(-0.856, -0.013)	100	0.043	32.2	0.171
	Population						
	Dyslipidemia	2	-0.074(-0.726, -0.577)	41.83	0.832	0.0	0.592
	Other diseases	6	-0.693(-1.246, -0.141)	52.58	0.016	37.7	0.155
	Dosage (g/day)						
	<0.9	2	-0.363(-1.019, 0.292)	41.35	0.277	86.3	0.686
	1	3	-0.625(-1.313, 0.063)	37.50	0.075	0.0	0.416
	1.5	3	-0.236(-1.153, 0.680)	21.15	0.613	0.0	0.007
	Duration(day)						
	84	2	-1.514(-2.731, -0.296)	11.99	0.015	65.3	0.090
	90	4	-0.256(-0.774, 0.262)	66.14	0.333	23.3	0.271
	≥112	2	-0.383(-1.284, 0.519)	21.87	0.405	0.0	0.824
TC (mmol/L)							
	Overall	7	-0.451(-0.631, -0.271)	100	<0.001	77.4	<0.001
	Population						
	Dyslipidemia	3	-0.373(-0.452, -0.295)	47.42	<0.001	0.0	0.381
	Other diseases	4	-0.467(-0.864, -0.070)	52.58	0.021	85.7	<0.001
	Dosage (g/day)						
	<0.9	2	-0.237(-0.509, -0.035)	32.67	0.087	65.7	0.088
	1	4	-0.515(-0.801, -0.229)	51.07	<0.001	71.2	0.015
	1.5	1	-0.700(-0.914, -0.486)	16.26	<0.001	0.0	<0.001
	Duration(day)						
	84	3	-0.341(-0.617, -0.064)	40.06	0.016	62.2	0.071
	90	3	-0.632(-0.984, -0.280)	48.82	<0.001	89.5	<0.001
	≥112	1	-0.150(-0.521, 0.221)	11.12	0.429	0.0	<0.001
FPG (mmol/L)							
	Overall	6	-0.515(-0.847, -0.183)	100	0.002	89.8	<0.001
	Population						
	Dyslipidemia	1	-0.00(-0.162, 0.162)	18.41	1.00	0.0	<0.001
	Other diseases	5	-0.631(-0.869, -0.413)	81.59	<0.001	63.0	0.029
	Dosage (g/day)						
	<0.9	2	-0.381(-1.136, -0.373)	36.15	0.322	96.7	<0.001
	1	2	-0.587(-0.896, -0.277)	29.44	<0.001	13.3	0.283
	1.5	2	-0.568(-1.088, -0.049)	34.41	0.032	87.3	0.005
	Duration(day)						
	84	2	-0.797(-0.962, -0.631)	35.12	<0.001	0.0	0.724
	90	3	-0.307(-0.693, 0.079)	48.95	0.120	81.9	0.004
	≥112	1	-0.470(-0.816, -0.124)	15.92	0.008	0.0	<0.001

Nine RCTs (total sample size = 774 participants) evaluated the effect of berberine on HDL-C. The random-effects model showed no significant difference between berberine and placebo (WMD: 0.016 mmol/L; 95% CI: −0.045 to 0.078; *p* = 0.60), with substantial heterogeneity (I^2^ = 83.3%, *p* < 0.001) (Figure 3B). Subgroup analysis indicated that berberine improved HDL-C levels more effectively when the dose was 1.5 g/day, and the treatment duration was ≤90 days, compared to other subgroups. Heterogeneity decreased when stratified by dose and intervention duration (Table 3).

Eight RCTs (total sample size = 750 participants) assessed the effect of berberine on LDL-C. The random-effects model revealed a significant reduction in LDL-C levels with berberine intervention (WMD: −0.495 mmol/L; 95% CI: −0.714 to −0.276; *p* < 0.001), with substantial heterogeneity (I^2^ = 89.1%, *p* < 0.001) (Figure 3C). Subgroup analysis showed a more significant reduction in LDL-C when the treatment duration was ≤90 days compared to 112 days (Table 3).

Eight RCTs (total sample size = 684 participants) evaluated the effect of berberine on BMI. The fixed-effects model indicated that berberine significantly reduced BMI (WMD: −0.435 kg/m^2^; 95% CI: −0.856 to −0.013; *p* = 0.043), with a stable effect (I^2^ = 32.3%, *p* = 0.171) (Figure 3D). Subgroup analysis showed a more significant effect on BMI reduction when the treatment duration was 84 days compared to other subgroups (Table 3).

Seven RCTs (total sample size = 707 participants) assessed the effect of berberine on TC. The random-effects model revealed a significant reduction in TC levels with berberine (WMD: −0.451 mmol/L; 95% CI: −0.631 to −0.271; *p* < 0.001), with substantial heterogeneity (I^2^ = 77.4%, *p* < 0.001) (Figure 3E). Subgroup analysis showed a greater effect on TC reduction when the treatment duration was ≤90 days. Heterogeneity decreased when stratified by treatment duration (Table 3).

Six RCTs (total sample size = 578 participants) evaluated the effect of berberine on FPG. The random-effects model revealed a significant reduction in FPG with berberine (WMD: −0.515 mmol/L; 95% CI: −0.847 to −0.183; *p* = 0.002), with substantial heterogeneity (I^2^ = 89.8%, *p* < 0.001) (Figure 3F). Subgroup analysis indicated a greater effect on FPG reduction with a treatment duration of 84 or 112 days compared to other subgroups. Heterogeneity decreased when stratified by population subgroups (Table 3).

Four RCTs (total sample size = 396 participants) reported on the effect of berberine on WC. The fixed-effects model showed a significant reduction in WC levels with berberine (WMD: −3.270 cm; 95% CI: −4.818 to −1.722; *p* < 0.001), with a stable effect (I^2^ = 0.00%, *p* = 0.484) (Figure 3G).

Three RCTs (total sample size = 214 participants) evaluated the effect of berberine on SBP. The random-effects model showed no significant reduction in SBP (WMD: −0.879 mmHg; 95% CI: −7.223 to 5.464; *p* = 0.786), with significant heterogeneity (I^2^ = 68.8%, *p* = 0.041) (Figure 3H).

Three RCTs (total sample size = 214 participants) evaluated the effect of berberine on DBP. The random-effects model showed no significant reduction in DBP (WMD: 0.424 mmHg; 95% CI: −4.894 to 5.741; *p* = 0.876), with significant heterogeneity (I^2^ = 73.7%, *p* = 0.022) (Figure 3I).

Three RCTs (total sample size = 292 participants) evaluated the effect of berberine on 2hOGTT. The fixed-effects model revealed a significant reduction in 2hOGTT levels with berberine (WMD: −1.606 mmol/L; 95% CI: −1.891 to −1.321; *p* < 0.001), with a stable effect (I^2^ = 0.00%, *p* = 0.623) (Figure 3J).

The risk of bias assessment identified two low-quality studies (Pu et al., 2021; Ran, 2023), and we excluded them in an additional meta-analysis to assess the robustness of the results. For blood pressure and 2-h oral glucose tolerance test (2hOGTT), no high-risk studies were identified, and the findings remained stable. The additional meta-analysis demonstrated that berberine remains its therapeutic effects on the following MetS parameters after excluding the two low-quality studies: TG (WMD: −0.354 mmol/L; 95% CI: −0.562 to −0.146; *p* < 0.01), HDL-C (WMD: 0.021 mmol/L; 95% CI: −0.045 to 0.087; *p* = 0.538), FPG (WMD: −0.460 mmol/L; 95% CI: −0.825 to −0.095; *p* = 0.014), and WC (WMD: −3.044 cm; 95% CI: −4.769 to −1.320; *p* < 0.01). Additionally, a change was observed in BMI (WMD: −0.302 kg/m^2^; 95% CI: −0.736 to 0.132; *p* = 0.173), which indicates a moderate attenuation in berberine’s effect on BMI.

### 3.5 Sensitivity analysis

**FIGURE 4:**
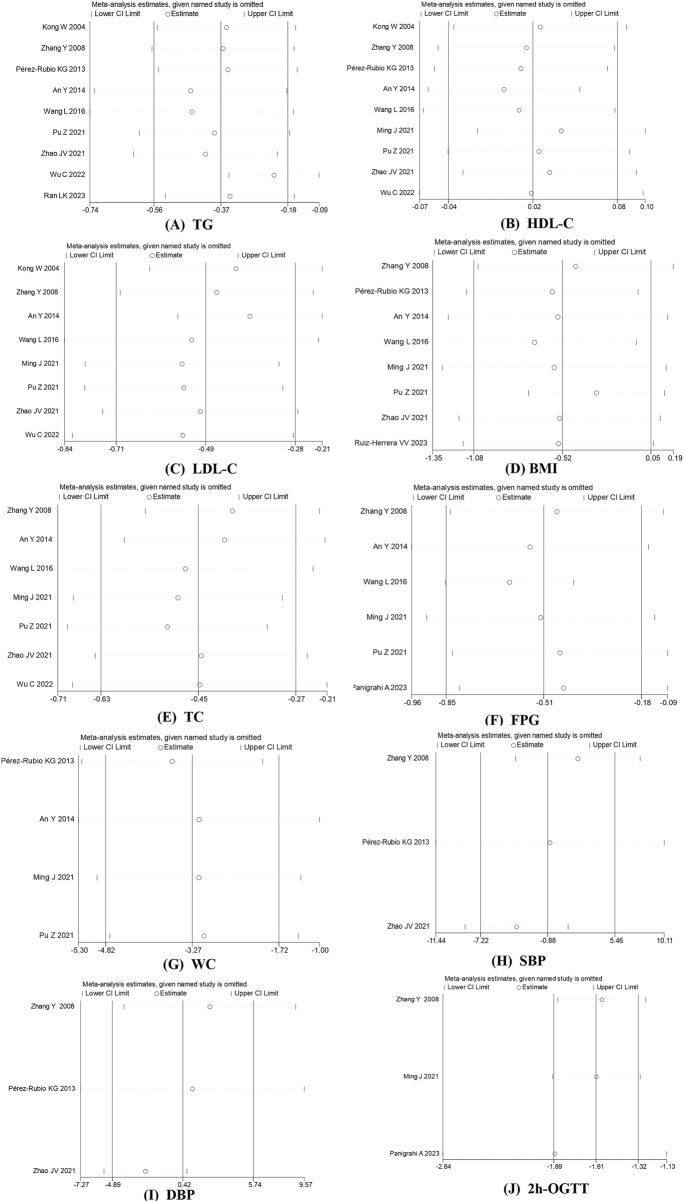
Sensitivity analysis was performed by removing each study in turn to determine the impact of each study on the overall effect size. **(A)** TG; **(B)** HDL-C **(C)** LDL-C; **(D)** BMI **(E)** TC; **(F)** FPG **(G)** WC; **(H)** SBP **(I)** DBP; **(J)** 2h-OGTT.

To assess the impact of each study on the overall effect size, each study was excluded one at a time, and the total effect size was re-analyzed. We found that none of the individual studies significantly affected the overall effect size for TG, HDL-C, LDL-C, TC, BMI, FPG, SBP, WC, or DBP. However, the sensitivity analysis for 2hOGTT indicated that the overall estimate was influenced by the study by Panigrahi A et al. (WMD: −1.884; 95% CI: −2.640 to −1.128) (Panigrahi and Mohanty, 2023), as shown in Figure 4. After excluding low-quality studies, the results of the above indicators remained stable (Supplementary Figure S3.5).

### 3.6 Meta-regression

**FIGURE 5:**
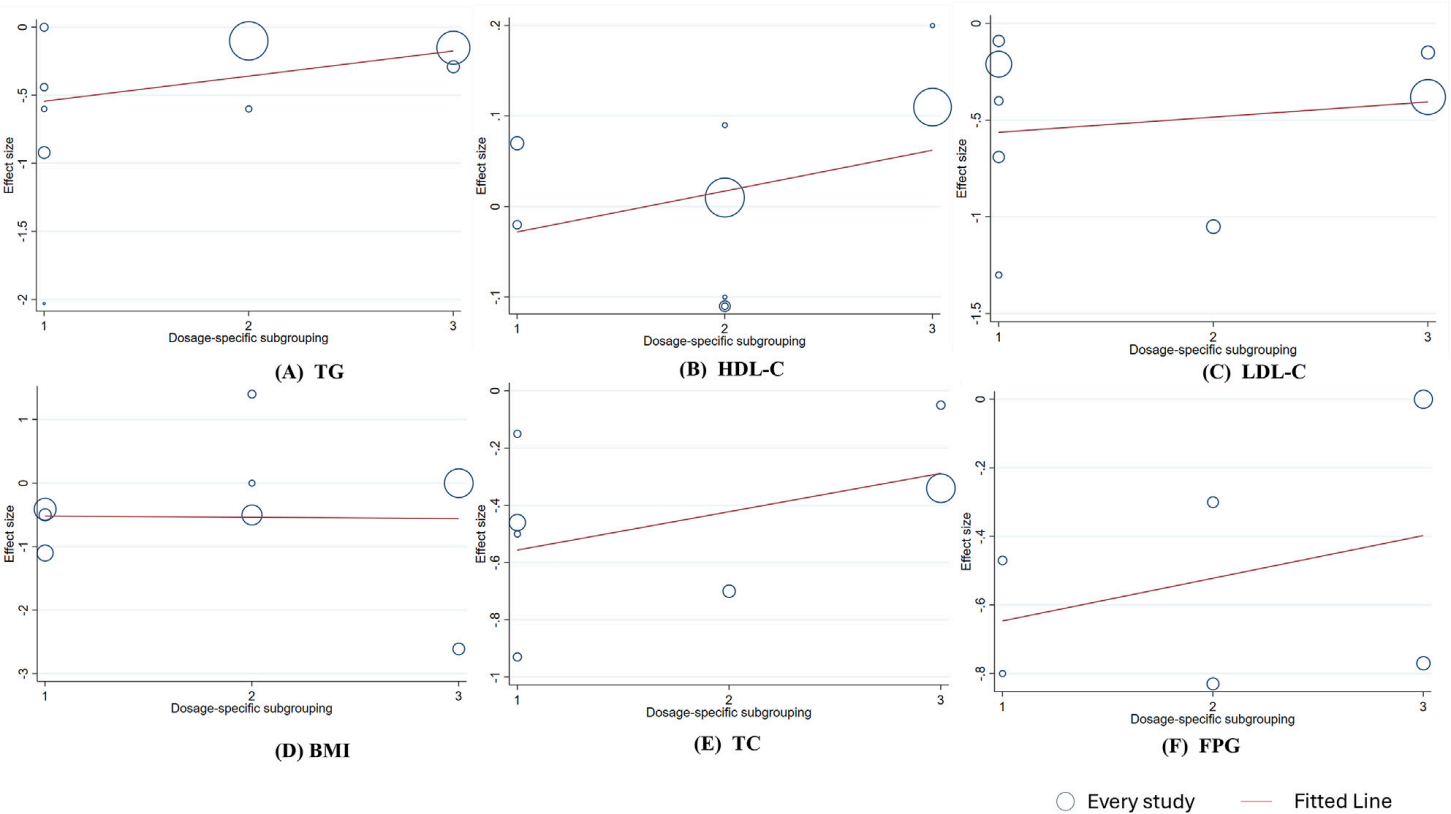
Meta-regression analysis between berberine dosage and mean difference **(A)** TG **(B)** HDL-C; **(C)** LDL-C **(D)** BMI; **(E)** TC; **(F)** FPG. 1 ≤ 0.9 g/day, 2 = 1 g/ gay, 3 ≥ 1.5 g/day. The size of the bubble represents the weight of each individual study.

**FIGURE 6:**
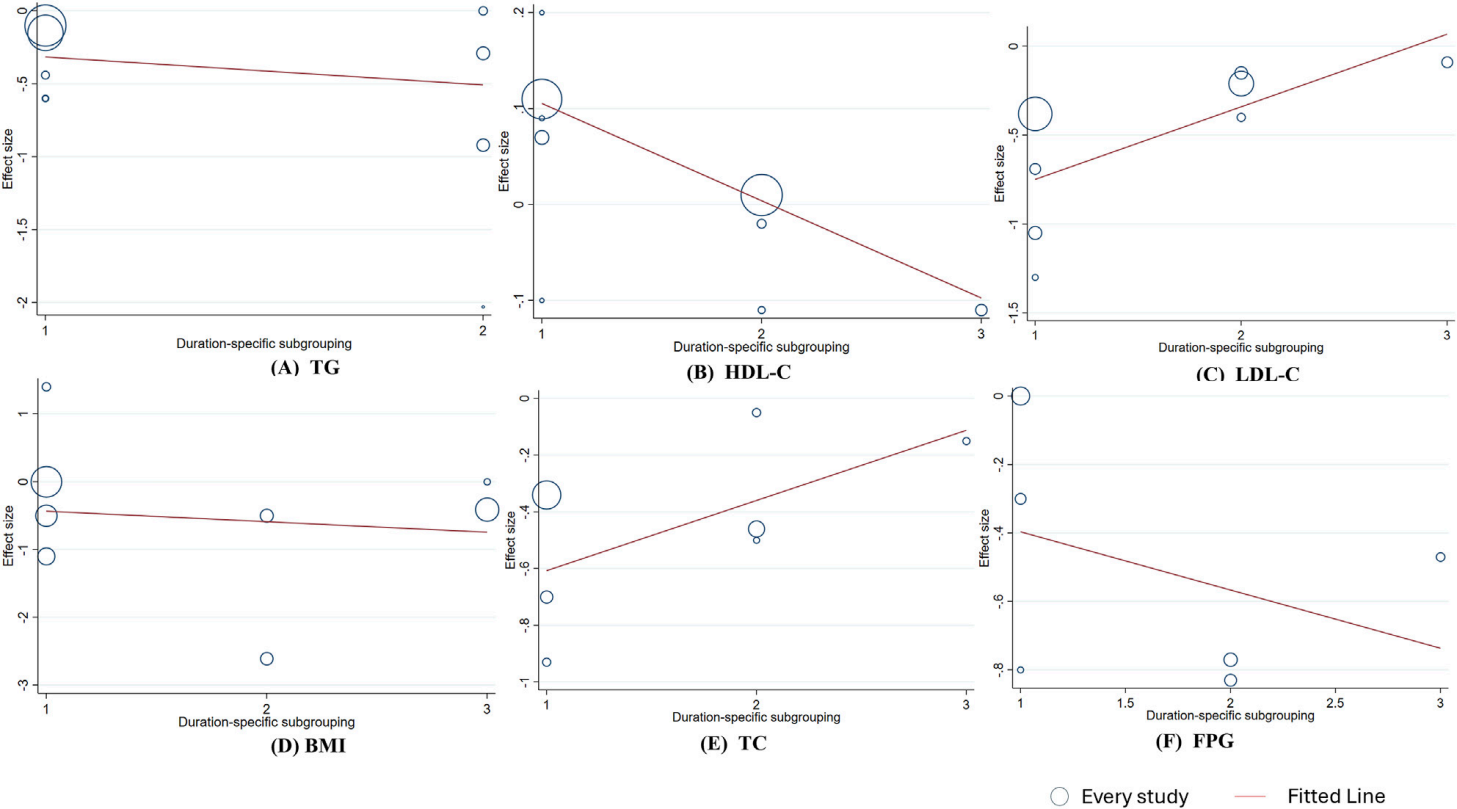
Meta-regression analysis between berberine duration and mean difference **(A)** TG **(B)** HDL-C; **(C)** LDL-C **(D)** BMI; **(E)** TC; **(F)** FPG. 1 = day, 2 = 90 days, 3 ≥ 112 days. The size of the bubble represents the weight of each individual study.

**FIGURE 7:**
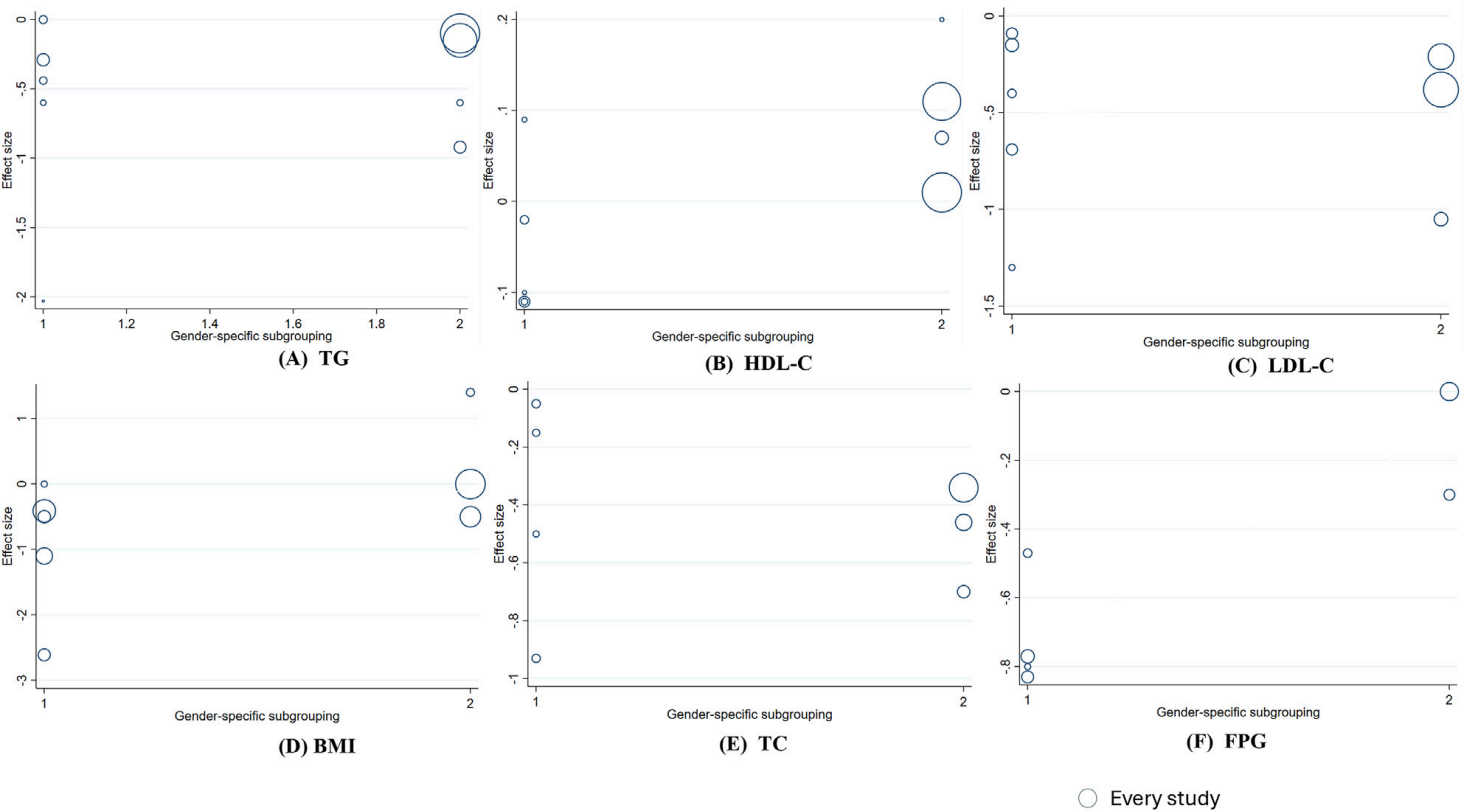
Meta-regression analysis between berberine gender and mean difference **(A)** TG **(B)** HDL-C; **(C)** LDL-C **(D)** BMI; **(E)** TC; **(F)** FPG. 1 = male predominant, 2 = female-predominant. The size of the bubble represents the weight of each individual study.

**FIGURE 8:**
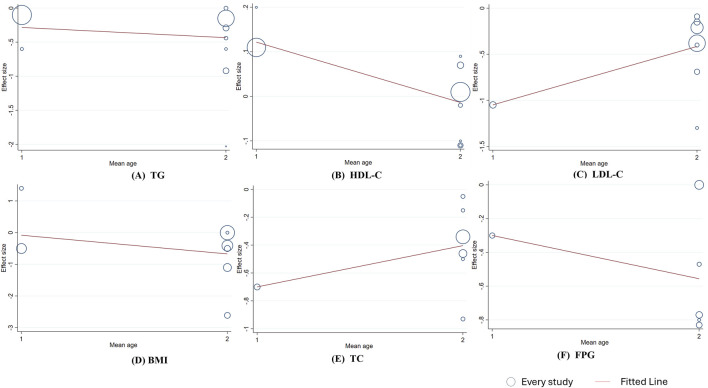
Meta-regression analysis between berberine gender and mean difference **(A)** TG **(B)** HDL-C; **(C)** LDL-C **(D)** BMI; **(E)** TC; **(F)** FPG. 1 = 20-39 years, 2 = 40-59 years female-predominant. The size of the bubble represents the weight of each individual study.

To assess the reliability of the study and identify factors contributing to heterogeneity, linear regression analysis was conducted for indicators with ≥6 studies to determine whether the results were influenced by berberine (berberine) dosage and intervention duration. The analysis found no significant linear relationship between changes in TG, TC, BMI, FPG, HDL-C, or LDL-C and the intervention dose (*p*_linear_ > 0.05, Figure 5). However, meta-regression showed a significant negative linear relationship between changes in HDL-C levels and intervention duration (HDL-C: Coef. = −0.101, *p*_linear_ < 0.001), and a significant positive linear relationship between changes in LDL-C levels and intervention duration (Coef. = 0.408, *p*_linear_ = 0.038). The linear relationship between intervention duration and the effects on TG, TC, BMI, and FPG was not significant. (Figure 6). In addition, a significant negative linear relationship was observed between TC and participants’ mean age (Coef. = −0.101, *p*_linear_ < 0.001). And FPG (Coef. = 0.617, *p* = 0.013) and HDL-C (Coef. = 0.131, *p* = 0.033) were significantly associated with sex stratification. No significant associations were found between mean age or sex stratification and the other outcomes (Figures 7,8).

### 3.7 Publication bias

Publication bias was assessed using funnel plots, along with Egger’s and Begg’s tests. Visual inspection of the funnel plots for all outcomes (Supplementary Figure S3.6) indicated slight asymmetry. However, according to the results of the Egger’s and Begg’s tests for HDL-C, LDL-C, TC, BMI, FPG, SBP, WC, DBP, and 2hOGTT, no significant publication bias was detected. For TG, Egger’s test yielded a p -value of 0.041, and Begg’s test yielded a p -value of 0.048, both indicating significant differences (Supplementary Figure S3.7; Supplementary Figure S3.8). Therefore, the Trim and Fill method was applied for correction, but the TG results remained unchanged, demonstrating robust effects (WMD: −0.367 mmol/L; 95% CI: −0.560 to −0.175; *p* < 0.01).

## 4 Discussion

This study analyzed 12 RCTs with a total of 889 patients, providing a comprehensive evaluation of the effects of berberine on MetS components, including FPG, TG, HDL-C, blood pressure, and WC. Berberine significantly reduced FPG, TG, and WC. However, its effects on blood pressure and HDL-C were not significant. Additionally, berberine significantly affected LDL-C, TC, BMI, and 2hOGTT. Short-term intervention (≤90 days) is more effective than long-term treatment on HDL-C and LDL-C, and there was no significant difference in safety between berberine and placebo.

In a recent meta-analysis of a randomized placebo-controlled trial, the efficacy of berberine on blood lipid profiles was evaluated (Blais et al., 2023). Berberine was found to significantly reduce TG, LDL-C, and TC concentrations, consistent with our findings. In addition, their study also reported a significant increase in HDL-C level in the overall population, a result that remains significant in the female subgroup but not in the male subgroup. The male participant group in our study (348 males/298 females) is larger, which may explain the lack of a significant increase in HDL-C in our overall analysis. Furthermore, a 2023 meta-analysis (Xie et al., 2022) reported that berberine intervention significantly reduced FPG and 2hOGTT levels; however, this meta-analysis only included T2DM patients, while our meta-analysis evaluated berberine’s effect in a broader population. Zamani et al. (2022) found improvements in BMI and WC with berberine. Our findings further suggest that berberine’s therapeutic effect can be better. Additionally, some studies (Lan et al., 2015; Zamani et al., 2022) found that berberine significantly reduced SBP but had no significant effect on DBP. The results for SBP in our study are different, likely because our study focused on specific berberine and used a more rigorous randomized placebo-controlled trial, while the other study used Berberis plant extracts with a blank control group (Zamani et al., 2022). Meanwhile, blood pressure can be influenced by various factors, leading to differing effects across populations (Zhang et al., 2008; Perez-Rubio et al., 2013), and the mechanisms behind these effects require further investigation.

In the subgroup analysis, the heterogeneity of TC in the hyperlipidemia group was lower, further supporting the stable effect of berberine on TC. Meanwhile, the effect of berberine on FPG was more pronounced in the non-hyperlipidemic population. These results consistently suggest that berberine primarily impacts abnormal indicators and may have a smaller effect on normal indicators. In the subgroup analyses for HDL-C, TC, LDL-C, and BMI, no significant treatment effects were observed when the intervention duration exceeded 112 days. Meta-regression also indicated that shorter-term berberine intervention (≤90 days) was more effective than longer-term treatment in terms of HDL-C and LDL-C. These findings support that berberine may be more effective in short-term treatment lasting ≤90 days, possibly due to drug tolerance or metabolic adaptation with extended treatment (Cao et al., 2021). An exception was that berberine had no significant effect on FPG in the 90-day subgroup. Further analysis suggested that this was likely due to most participants in this subgroup having normal blood glucose levels, as berberine does not significantly affect normal values for these indicators (Zhang et al., 2008; An et al., 2014; Wang et al., 2016b). Therefore, the strength of berberine’s therapeutic effects may be influenced by the characteristics of the study population. Our analysis included diverse populations, such as individuals with dyslipidemia, T2DM, and PCOS. Treatment responses may differ across these subgroups. For instance, our subgroup analysis revealed that berberine had a more pronounced effect on lipid parameters in participants with dyslipidemia (e.g., the pooled effect of TG in the dyslipidemia population was −0.513 mmol/L and that of other diseases was −0.264 mmol/L), whereas its glucose-lowering benefits were more evident in non-dyslipidemic populations (the pooled effect of FPG in the dyslipidemia population was −0.00 mmol/L and that of other diseases was −0.631 mmol/L). This suggests that berberine therapy may be most effective when targeted toward specific types of MetS. These findings highlight the importance of considering population heterogeneity when evaluating the efficacy of therapeutic agents.

In addition, subgroup analyses and meta-regression revealed that mean age may be a source of heterogeneity for TC. The therapeutic effect of berberine on TC was more pronounced in younger individuals compared to older ones, supporting the observation that younger patients tend to respond more favorably to pharmacological treatments (Pennybaker et al., 2021). Furthermore, improvements in FPG appeared to be sex-specific, with studies involving predominantly female participants yielding significantly different results compared to those with predominantly male participants. This finding aligns with previous evidence suggesting sex-related differences in berberine’s glycemic control efficacy (Zhao et al., 2023).

A key limitation of our analysis was the substantial heterogeneity observed across several outcomes, including TG, HDL-C, LDL-C, and FPG. While our subgroup and meta-regression analyses partially explained this variability, some heterogeneity remained unexplained. This may be attributed to methodological differences across studies or the inherent complexity of treating metabolic syndrome indicators. Such heterogeneity ultimately limits the precision of our efficacy estimates for berberine and suggests that treatment responses may vary among different patient populations.

Berberine can cause mild adverse effects, such as nausea, vomiting, and constipation, although these are not commonly seen in clinical practice (Yang et al., 2023). Our study found that berberine showed no significant difference compared to placebo in terms of adverse effects. However, two of the 12 included studies lacked safety data, introducing potential limitations to our safety analysis. Therefore, the safety outcomes of berberine necessitate further validation through additional rigorously designed studies.

Although this meta-analysis adhered strictly to the Cochrane methodology for literature retrieval and screening, several important limitations should be considered when interpreting our results and translating them into clinical practice: (1) The included studies exhibited methodological shortcomings (Figure 2). Although sensitivity and additional analyses indicated that these did not substantially alter the main findings, the possibility of random bias and residual confounding cannot be entirely excluded. These limitations warrant cautious interpretation of the results. (2) The study populations varied across trials, and population-specific responses to berberine should be considered. (3) Due to the limited number of studies, subgroup analyses for WC, SBP, DBP, and 2hOGTT could not be performed, which may affect the robustness of these specific outcomes. (4) Safety data were incomplete in several studies; therefore, clinicians should exercise caution when using berberine as monotherapy or adjunctive therapy and ensure close monitoring for both efficacy and adverse events.

Currently, most available evidence stems from randomized controlled trials focusing on individual components of metabolic syndrome, with limited data regarding treatment duration and long-term efficacy. This meta-analysis suggests that berberine improves glycemic and lipid parameters, with short-term intervention demonstrating comparatively greater efficacy than long-term administration for modulating HDL-C and LDL-C levels. Nevertheless, the current evidence base is insufficient to claim syndrome-level remission. Large-scale, multicenter trials featuring standardized designs and rigorous reporting are still required in metabolic syndrome populations. Such studies would clarify berberine’s true therapeutic role, strengthen the evidence for its use in managing metabolic syndrome components, and identify optimal intervention time and duration for sustained clinical benefit.

## 5 Conclusion

Our analysis indicates that berberine significantly reduces TG, LDL-C, TC, BMI, WC, FPG, and 2hOGTT levels, with a favorable safety profile. Although no overall effect was observed on HDL-C, SBP, or DBP, short-term treatment (≤90 days) has better effects than long-term treatment for HDL-C and LDL-C. These findings suggest that berberine may offer significant benefits in improving lipid and glucose metabolism indices of MetS. Current evidence is insufficient to claim syndrome-level remission; future large-scale, multicenter studies and accurately reported studies in the metabolic syndrome population are needed to clarify the effects of berberine on metabolic syndrome diseases.

## Data Availability

The original contributions presented in the study are included in the article/Supplementary Material, further inquiries can be directed to the corresponding author.

## Abbreviations

MetS, Metabolic syndrome; CI, confidence interval; FPG, fasting plasma glucose; HDL-C, high-density lipoprotein cholesterol; SBP, systolic blood pressure; BMI, body mass index; T2DM, type 2 diabetes; RoB 2, Risk of Bias Tool version 2; PCOS, polycystic ovary syndrome; ALT, glutamic-pyruvic transaminase; GGT, γ-glutamyl transferase; Cr, serum creatinine; PICOS, Participants, Intervention, Comparison, Outcomes, and Study Design; WMD, Weighted mean difference; TG, Triglycerides; WC, waist circumference; LDL-C, low-density lipoprotein cholesterol; DBP, diastolic blood pressure; 2h-OGTT, 2-h oral glucose tolerance test; RCT, Randomized controlled trial; IFG, Impaired fasting glucose; HIV, human immunodeficiency virus; AST, aspartate aminotransferase; ALP, alkaline phosphatase; SE, serum electrolyte.
