# Supplementary material for: Efficacy and safety of berberine on the components of metabolic syndrome: a systematic review and meta-analysis of randomized placebo-controlled trials
Source: Front Pharmacol. 2025 Jul 16;16:1572197. doi: 10.3389/fphar.2025.1572197 (PMC12307485; doi:10.3389/fphar.2025.1572197)
Supplement: Supplementary file 1 [file DataSheet1.docx]

Supplementary Material

# Search strategies for databases

| Database | Search | Search Strings |
| --- | --- | --- |
| PubMed | #1 | (berberine[Title/Abstract]) OR(Umbellatine) |
|  | #2 | (Metabolic Syndromes[Title/Abstract]) OR(Reaven Syndrome X) OR(Syndrome X, Reaven) OR(Insulin Resistance Syndrome X) OR(Metabolic Cardiovascular Syndrome) OR(Cardiovascular Syndrome, Metabolic) OR(Cardiovascular Syndromes, Metabolic) OR(Syndrome X, Insulin Resistance) OR(Syndrome, Metabolic X) OR(Dysmetabolic Syndrome X) OR(Syndrome X, Dysmetabolic) OR(Syndrome X, Metabolic) OR(Cardiometabolic Syndromes) OR(Metabolic Syndrome) OR(MetS) OR(MS) OR(Hyperlipidemia) OR(Hyperlipemia) OR(Hyperlipemias) OR(Lipidemia) OR(Lipidemias) OR(Lipemia) OR(Lipemias) OR(Hyperlipidemias) OR(High Blood Pressure) OR(High Blood Pressures) OR(Hypertension) OR(Hyperglycemias) OR(Hyperglycemia) OR(Obesity) OR(obese) OR(overweight) OR(waist) OR(waist circumference)OR(waist circumferences) |
|  | #3 | (randomized clinical trial) OR(randomized controlled trial) OR(trial) OR(clinical trial) OR(randomized) OR(random) OR(randomly) OR(placebo) OR(RCT) OR(Intervention) OR(Controlled trial) OR(Randomised) OR(Cross-over) OR(Crossover) OR(Blinded) OR(Parallel) |
|  | #4 | (((berberine[Title/Abstract]) OR(Umbellatine)) AND ((Metabolic Syndromes[Title/Abstract]) OR(Reaven Syndrome X) OR(Syndrome X, Reaven) OR(Insulin Resistance Syndrome X) OR(Metabolic Cardiovascular Syndrome) OR(Cardiovascular Syndrome, Metabolic) OR(Cardiovascular Syndromes, Metabolic) OR(Syndrome X, Insulin Resistance) OR(Syndrome, Metabolic X) OR(Dysmetabolic Syndrome X) OR(Syndrome X, Dysmetabolic) OR(Syndrome X, Metabolic) OR(Cardiometabolic Syndromes) OR(Metabolic Syndrome) OR(MetS) OR(MS) OR(Hyperlipidemia) OR(Hyperlipemia) OR(Hyperlipemias) OR(Lipidemia) OR(Lipidemias) OR(Lipemia) OR(Lipemias) OR(Hyperlipidemias) OR(High Blood Pressure) OR(High Blood Pressures) OR(Hypertension) OR(Hyperglycemias) OR(Hyperglycemia) OR(Obesity) OR(obese) OR(overweight) OR(waist) OR(waist circumference)OR(waist circumferences))) AND ((randomized clinical trial) OR(randomized controlled trial) OR(trial) OR(clinical trial) OR(randomized) OR(random) OR(randomly) OR(placebo) OR(RCT) OR(Intervention) OR(Controlled trial) OR(Randomised) OR(Cross-over) OR(Crossover) OR(Blinded) OR(Parallel)) |
| Web of Science | #1 | TI=((berberine) OR(Umbellatine)) |
|  | #2 | TI=((Metabolic Syndromes) OR(Reaven Syndrome X) OR(Syndrome X, Reaven) OR(Insulin Resistance Syndrome X) OR(Metabolic Cardiovascular Syndrome) OR(Cardiovascular Syndrome, Metabolic) OR(Cardiovascular Syndromes, Metabolic) OR(Syndrome X, Insulin Resistance) OR(Syndrome, Metabolic X) OR(Dysmetabolic Syndrome X) OR(Syndrome X, Dysmetabolic) OR(Syndrome X, Metabolic) OR(Cardiometabolic Syndromes) OR(Metabolic Syndrome) OR(MetS) OR(MS) OR(Hyperlipidemia) OR(Hyperlipemia) OR(Hyperlipemias) OR(Lipidemia) OR(Lipidemias) OR(Lipemia) OR(Lipemias) OR(Hyperlipidemias) OR(High Blood Pressure) OR(High Blood Pressures) OR(Hypertension) OR(Hyperglycemias) OR(Hyperglycemia) OR(Obesity) OR(obese) OR(overweight) OR(waist) OR(waist circumference)OR(waist circumferences)) |
|  | #3 | ALL=((randomized clinical trial) OR(randomized controlled trial) OR(trial) OR(clinical trial) OR(randomized) OR(random) OR(randomly) OR(placebo) OR(RCT) OR(Intervention) OR(Controlled trial) OR(Randomised) OR(Cross-over) OR(Crossover) OR(Blinded) OR(Parallel)) |
|  | #5 | #1 AND #2 AND #3 |
|  | #6 | AB=((berberine) OR(Umbellatine)) |
|  | #7 | AB=((Metabolic Syndromes) OR(Reaven Syndrome X) OR(Syndrome X, Reaven) OR(Insulin Resistance Syndrome X) OR(Metabolic Cardiovascular Syndrome) OR(Cardiovascular Syndrome, Metabolic) OR(Cardiovascular Syndromes, Metabolic) OR(Syndrome X, Insulin Resistance) OR(Syndrome, Metabolic X) OR(Dysmetabolic Syndrome X) OR(Syndrome X, Dysmetabolic) OR(Syndrome X, Metabolic) OR(Cardiometabolic Syndromes) OR(Metabolic Syndrome) OR(MetS) OR(MS) OR(Hyperlipidemia) OR(Hyperlipemia) OR(Hyperlipemias) OR(Lipidemia) OR(Lipidemias) OR(Lipemia) OR(Lipemias) OR(Hyperlipidemias) OR(High Blood Pressure) OR(High Blood Pressures) OR(Hypertension) OR(Hyperglycemias) OR(Hyperglycemia) OR(Obesity) OR(obese) OR(overweight) OR(waist) OR(waist circumference)OR(waist circumferences)) |
|  | #8 | ALL=((randomized clinical trial) OR(randomized controlled trial) OR(trial) OR(clinical trial) OR(randomized) OR(random) OR(randomly) OR(placebo) OR(RCT) OR(Intervention) OR(Controlled trial) OR(Randomised) OR(Cross-over) OR(Crossover) OR(Blinded) OR(Parallel)) |
|  | #9 | (#6) AND (#7) AND (#8) |
|  | #10 | (#5) OR(#9) |
| EMBASE | #1 | (berberine:ab,ti OR umbellatine:ab,ti) AND ('metabolic syndromes':ab,ti OR 'reaven syndrome x':ab,ti OR 'syndrome x, reaven':ab,ti OR 'insulin resistance syndrome x':ab,ti OR 'metabolic cardiovascular syndrome':ab,ti OR 'cardiovascular syndrome, metabolic':ab,ti OR 'cardiovascular syndromes, metabolic':ab,ti OR 'syndrome x, insulin resistance':ab,ti OR 'syndrome, metabolic x':ab,ti OR 'dysmetabolic syndrome x':ab,ti OR 'syndrome x, dysmetabolic':ab,ti OR 'syndrome x, metabolic':ab,ti OR 'cardiometabolic syndromes':ab,ti OR 'metabolic syndrome':ab,ti OR mets:ab,ti OR ms:ab,ti OR hyperlipidemia:ab,ti OR hyperlipemia:ab,ti OR hyperlipemias:ab,ti OR lipidemia:ab,ti OR lipidemias:ab,ti OR lipemia:ab,ti OR lipemias:ab,ti OR hyperlipidemias:ab,ti OR 'high blood pressure':ab,ti OR 'high blood pressures':ab,ti OR hypertension:ab,ti OR hyperglycemias:ab,ti OR hyperglycemia:ab,ti OR obesity:ab,ti OR obese:ab,ti OR overweight:ab,ti OR waist:ab,ti OR 'waist circumference':ab,ti OR 'waist circumferences':ab,ti) |
|  | #2 | 'randomized clinical trial' OR 'randomized controlled trial' OR trial OR 'clinical trial' OR randomized OR random OR randomly OR placebo OR rct OR intervention OR 'controlled trial' OR randomised OR 'cross over' OR crossover OR blinded OR parallel |
|  | #3 | #1 AND #2 |
| Cochrane Library | #1 | (berberine) OR(Umbellatine) in Title Abstract Keyword |
|  | #2 | (Metabolic Syndromes) OR(Reaven Syndrome X) OR(Syndrome X, Reaven) OR(Insulin Resistance Syndrome X) OR(Metabolic Cardiovascular Syndrome) OR(Cardiovascular Syndrome, Metabolic) OR(Cardiovascular Syndromes, Metabolic) OR(Syndrome X, Insulin Resistance) OR(Syndrome, Metabolic X) OR(Dysmetabolic Syndrome X) OR(Syndrome X, Dysmetabolic) OR(Syndrome X, Metabolic) OR(Cardiometabolic Syndromes) OR(Metabolic Syndrome) OR(MetS) OR(MS) OR(Hyperlipidemia) OR(Hyperlipemia) OR(Hyperlipemias) OR(Lipidemia) OR(Lipidemias) OR(Lipemia) OR(Lipemias) OR(Hyperlipidemias) OR(High Blood Pressure) OR(High Blood Pressures) OR(Hypertension) OR(Hyperglycemias) OR(Hyperglycemia) OR(Obesity) OR(obese) OR(overweight) OR(waist) OR(waist circumference)OR(waist circumferences) in Title Abstract Keyword |
|  | #3 | (randomized clinical trial) OR(randomized controlled trial) OR(trial) OR(clinical trial) OR(randomized) OR(random) OR(randomly) OR(placebo) OR(RCT) OR(Intervention) OR(Controlled trial) OR(Randomised) OR(Cross-over) OR(Crossover) OR(Blinded) OR(Parallel) in All Text |
|  | #4 | #1 AND #2 AND #3 |
| China National Knowledge Infrastructure | #1 | (TI%黄连素 OR TI%小檗碱 OR AB%黄连素 OR AB%小檗碱) AND(TI%血脂过多 OR TI%血脂异常 OR TI%高脂血症 OR TI%脂血症 OR TI%血压过高 OR TI%高血压 OR TI%肥胖症 OR TI%肥胖 OR TI%体重 OR TI%超重 OR TI%多脂症 OR TI%腰围 OR TI%高血糖症 OR TI%血糖过高 OR TI%高糖血症 OR TI%高血糖 OR TI%高血脂症 OR TI%代谢综合征 OR TI%X 综合征 OR TI%胰岛素抵抗综合征 OR TI%抗胰岛素性综合征X OR TI%代谢不良X综合征 OR TI%Reaven X综合征 OR TI%代谢性心血管综合征 OR AB%血脂过多 OR AB%血脂异常 OR AB%高脂血症 OR AB%脂血症 OR AB%血压过高 OR AB%高血压 OR AB%肥胖症 OR AB%肥胖 OR AB%体重 OR AB%超重 OR AB%多脂症 OR AB%腰围 OR AB%高血糖症 OR AB%血糖过高 OR AB%高糖血症 OR AB%高血糖 OR AB%高血脂症 OR AB%代谢综合征 OR AB%X 综合征 OR AB%胰岛素抵抗综合征 OR AB%抗胰岛素性综合征X OR AB%代谢不良X综合征 OR AB%Reaven X综合征 OR AB%代谢性心血管综合征) AND FT%随机对照试验 |

# Supplementary material details the specific rationale for excluding adverse reaction data from this study.

Among the included studies, several trials exhibited missing or incomplete safety data, which may affect the comprehensiveness of the safety outcome synthesis. Wu et al. (2022) did not report any safety-related outcomes, likely because the study primarily focused on mechanistic investigations of berberine's effects on gut microbiota.

Wang et al. (2016) briefly described three mild adverse events (one case of transient headache and two cases of abdominal distension), but did not specify which treatment arm these events occurred in, rendering the data unusable for comparative analysis. Pérez-Rubio et al. (2013) only stated that "no significant adverse events were observed" without providing event counts, classifications, or group-specific details.

In addition, several studies reported safety data in a form that limited their inclusion in relative risk estimates. Specifically, Kong et al. (2004) did not report adverse events in the control group, and Pu et al. (2021) provided adverse event data for the control group only under the category of “any adverse event.” In such cases, these data were included in the calculation of absolute risk but excluded from relative risk analysis due to the lack of comparative information.

Some studies also lacked detailed definitions of specific adverse event categories. For example, Ming et al. (2021) reported "fecal abnormalities" without clear specification of included symptoms. Based on clinical convention, we interpreted this category to include diarrhea and constipation. Furthermore, this study reported four serious adverse events in the berberine group and three in the placebo group, without providing sufficient symptom details; given that no other included study reported serious adverse events, this outcome was not subjected to quantitative synthesis.

Where explicit safety set sizes were unavailable, we inferred them based on the number of participants who received at least one dose of the intervention and had at least one follow-up safety assessment. In cases where participants dropped out due to adverse effects, the safety population was taken to include both the completers and those who withdrew due to adverse events. When studies distinguished between treatment-related and non-treatment-related adverse events, only treatment-related events were included in our analysis. This applies specifically to Ming et al. (2021), where only adverse events explicitly attributed to berberine were counted. Additionally, when both the number of adverse event episodes and the number of affected individuals were reported, we used the number of individuals experiencing the event for synthesis. That is, adverse event count were based on the number of participants affected, not the total number of events.

Lastly, for studies reporting multiple related adverse symptoms (e.g., nausea and vomiting), we grouped them into broader categories (e.g., “gastrointestinal adverse events”) for synthesis purposes. While this may risk overestimation, our pooled analysis remained robust and did not show statistically significant differences between the berberine and placebo groups in any adverse event categories with sufficient data.

# Supplementary Figures


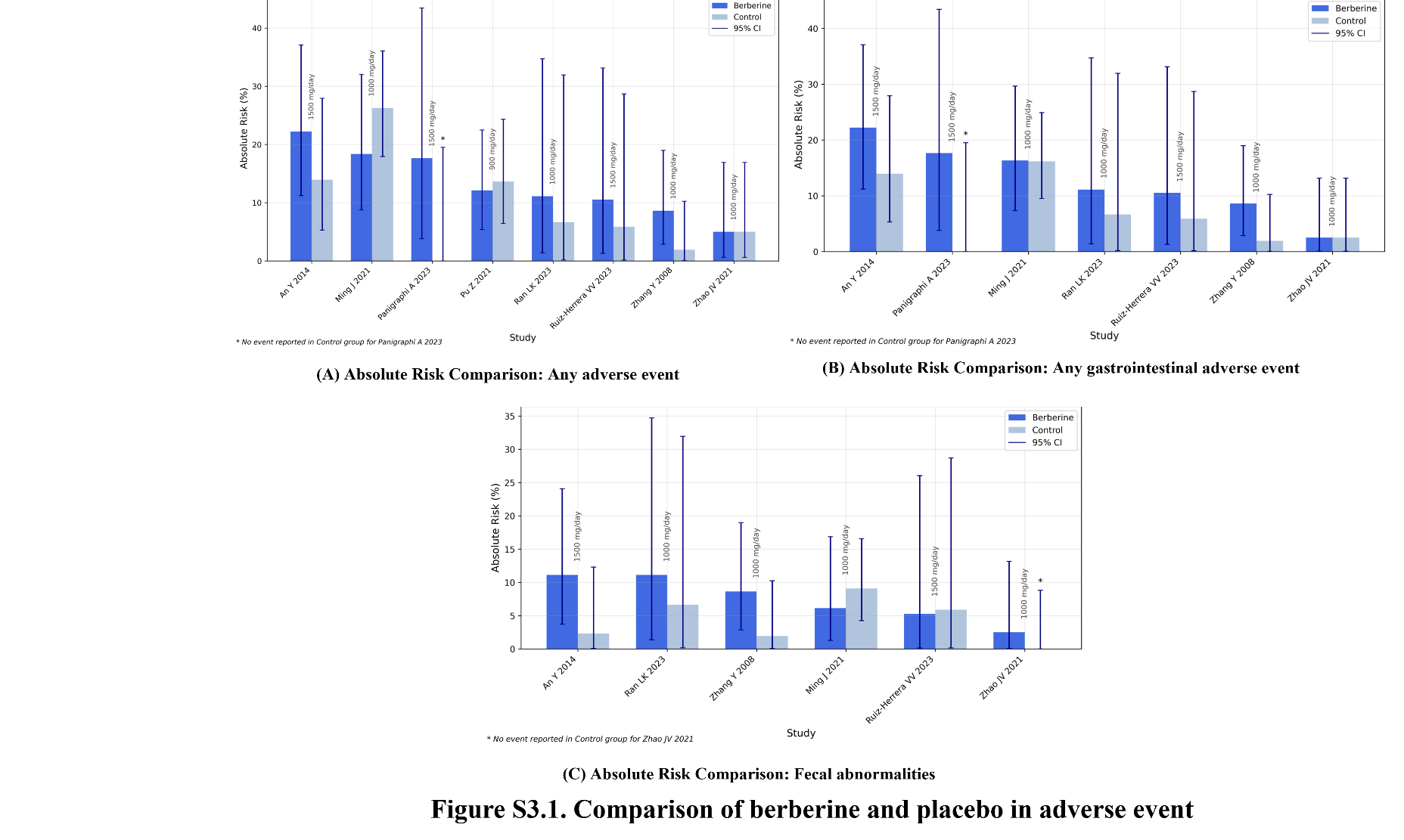


**Figure S3.1.** Comparison of berberine and placebo in adverse events.


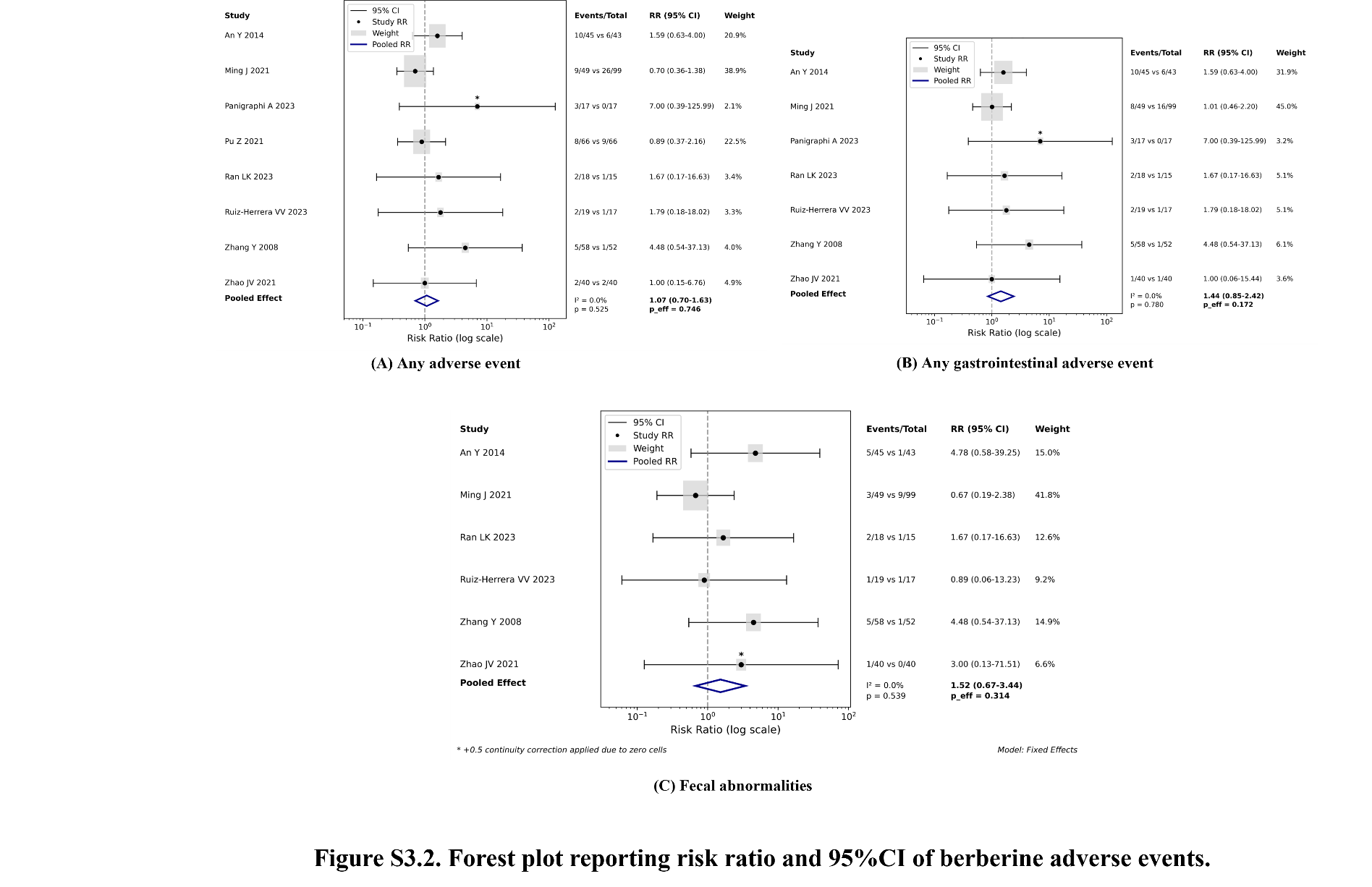


**Figure S3.2.** Forest plot reporting risk ratio and 95%CI of berberine adverse events.


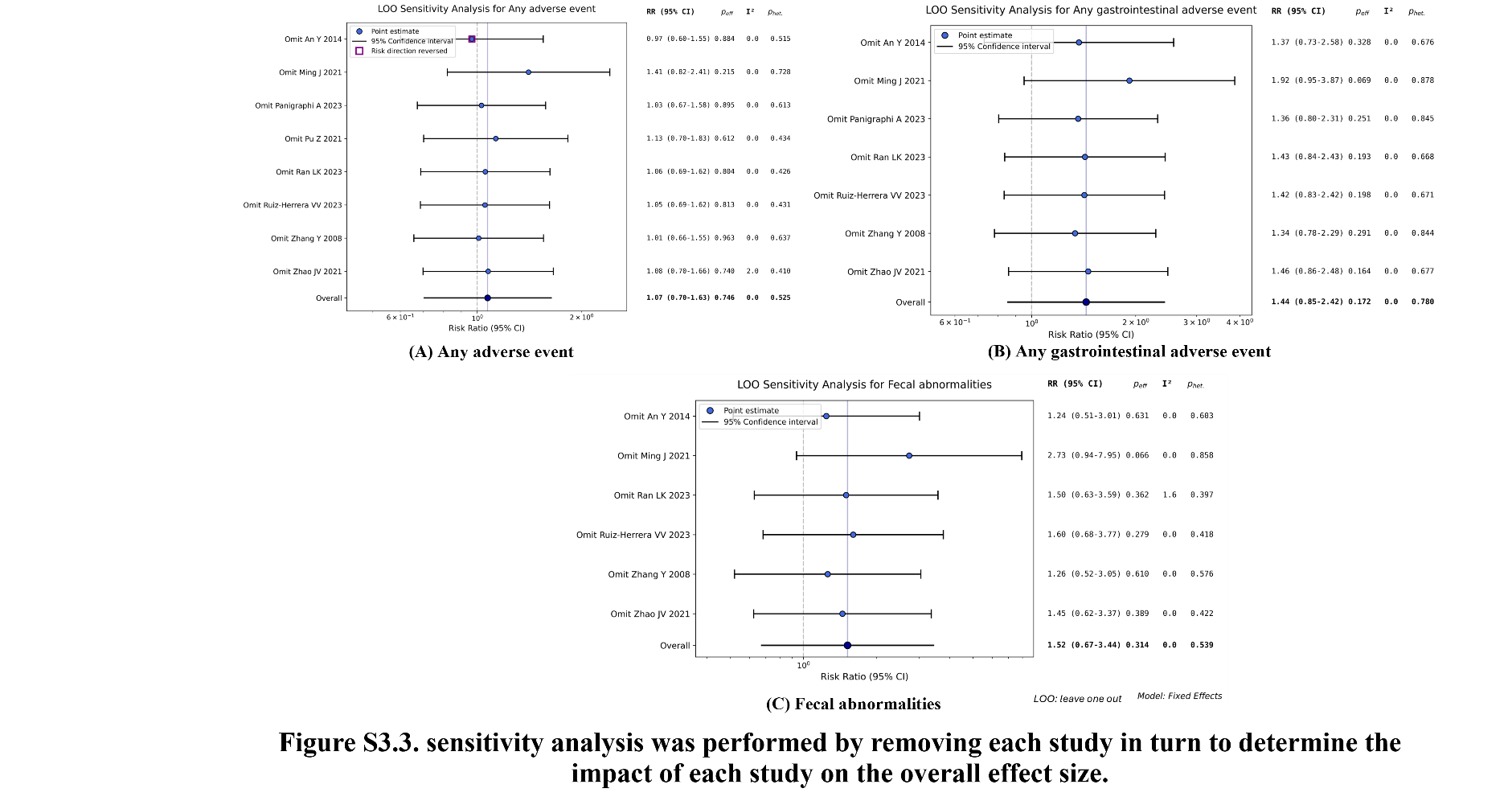


**Figure S3.3.** sensitivity analysis was performed by removing each study in turn to determine the impact of each study on the overall effect size.


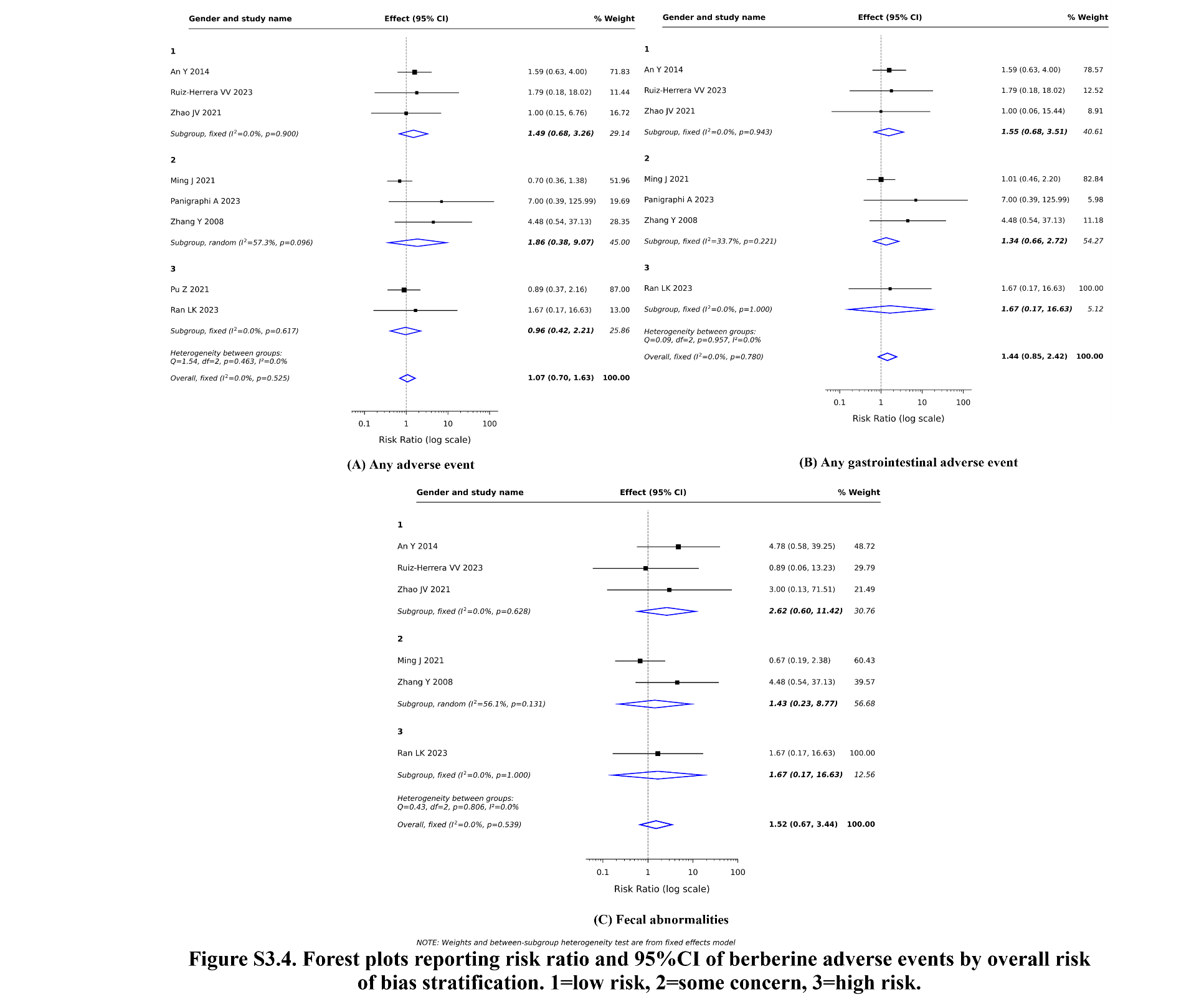


**Figure S3.4.** Forest plot reporting risk ratio and 95%CI of berberine adverse events by overall risk of bias stratification. 1=low risk, 2=some concern, 3=high risk.


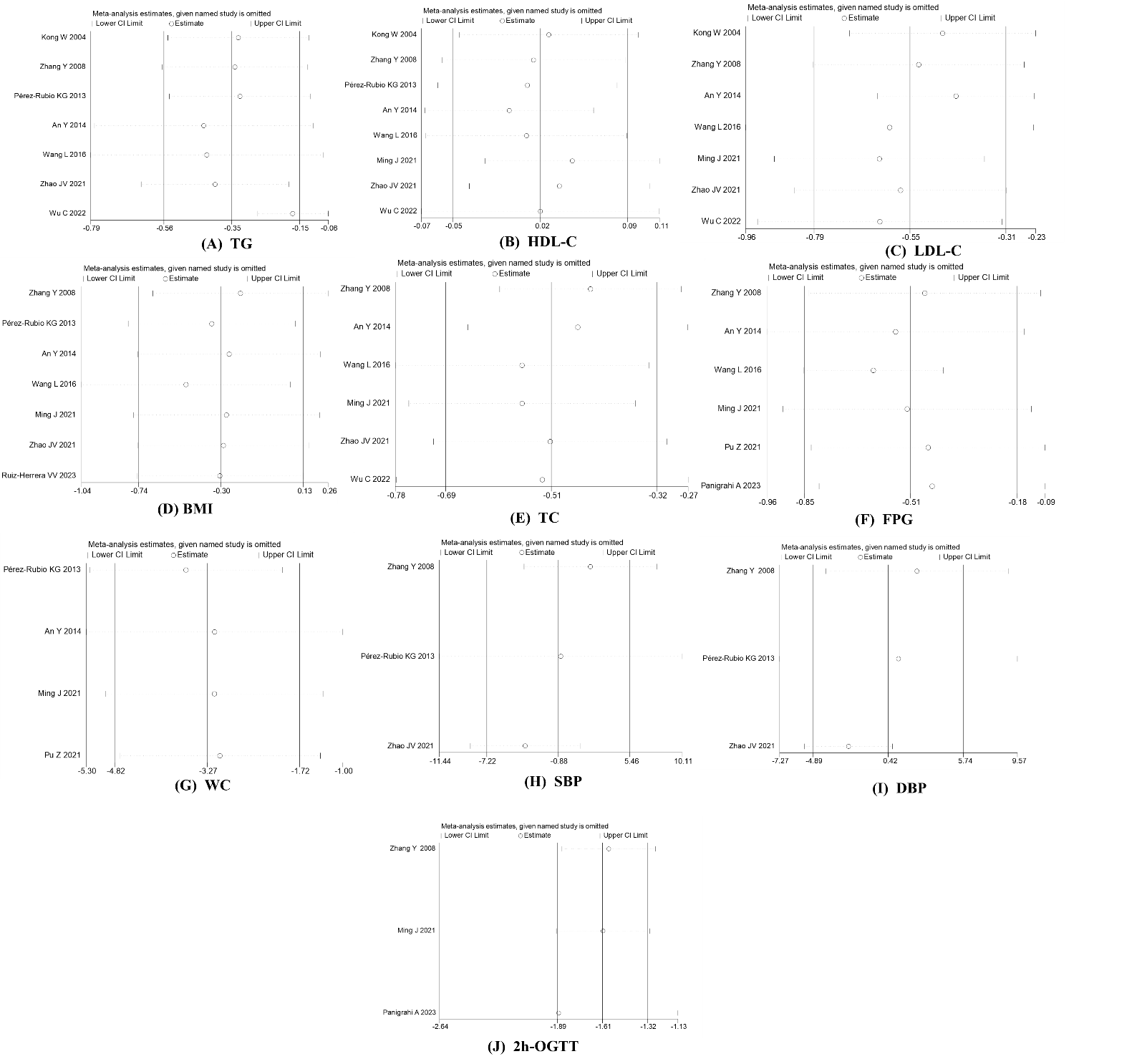


**Figure S3.5.** After removing low-quality studies, sensitivity analysis was performed by removing each study in turn to determine the impact of each study on the overall effect size.


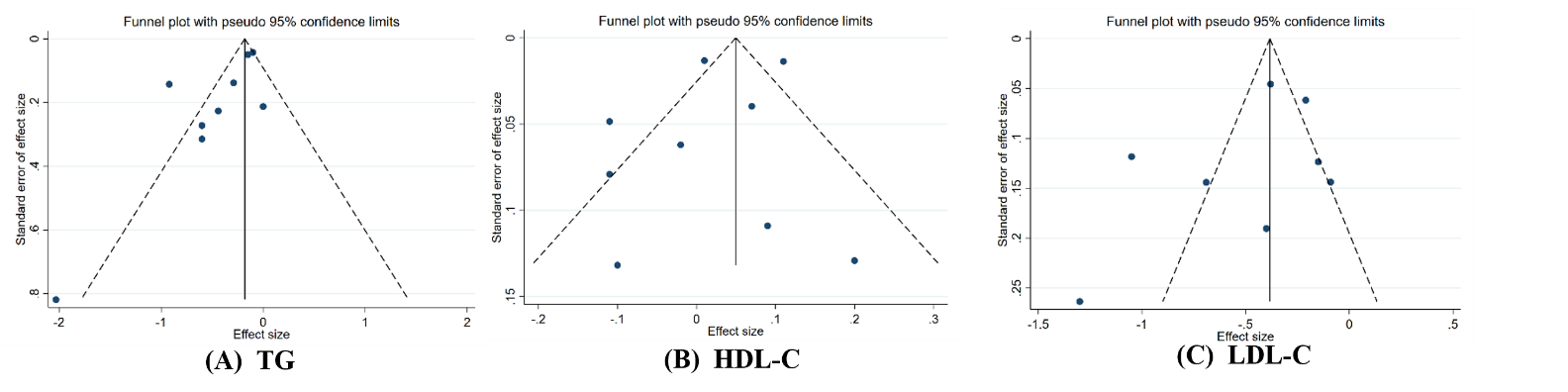


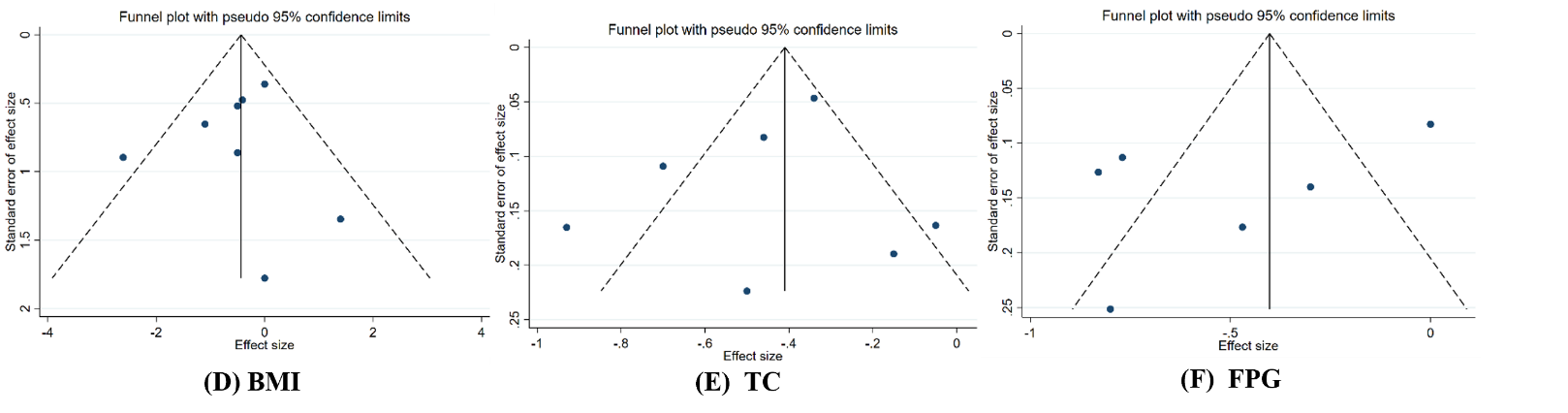


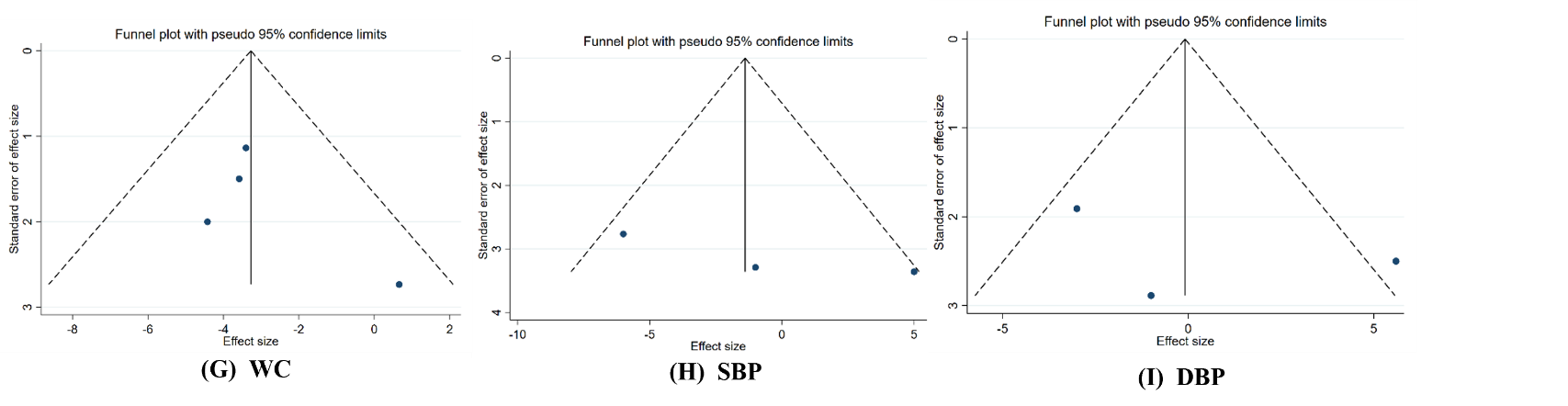


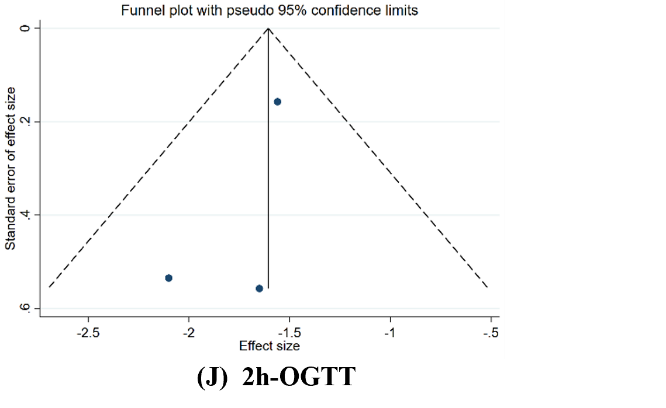


**Figure S3.6.** Funnel plot representing publication bias for the impact of berberine intake in (A) TG; (B) HDL-C; (C) LDL-C; (D) BMI; (E) TC; (F) FPG; (G) WC; (H) SBP; (I) DBP; (J) 2h OGTT.


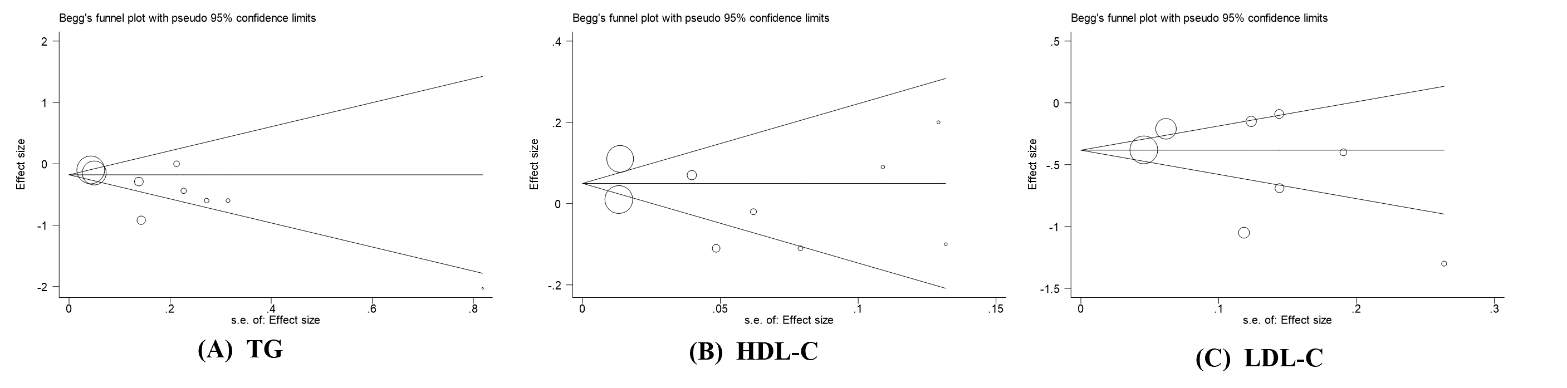


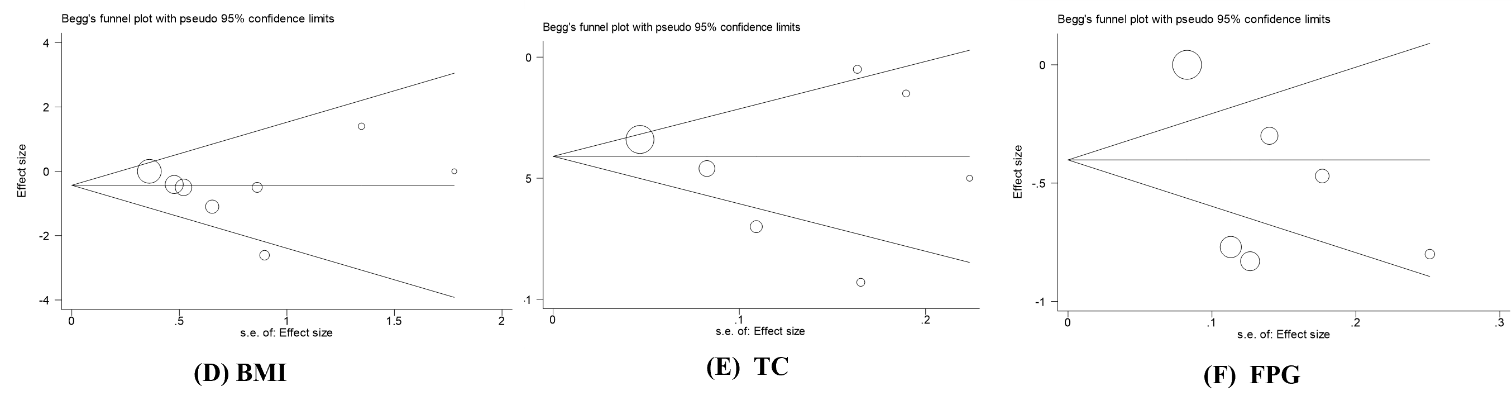


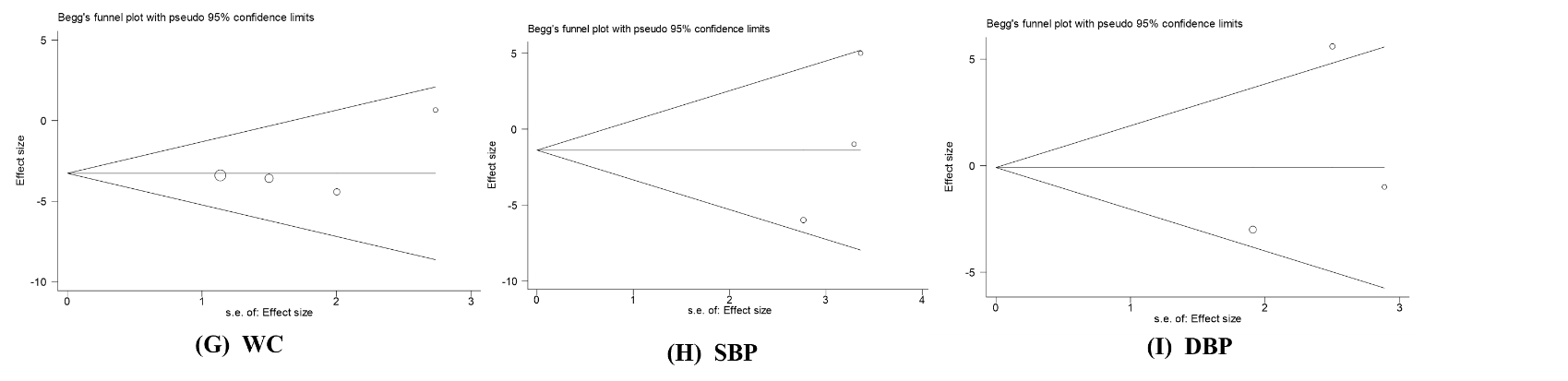


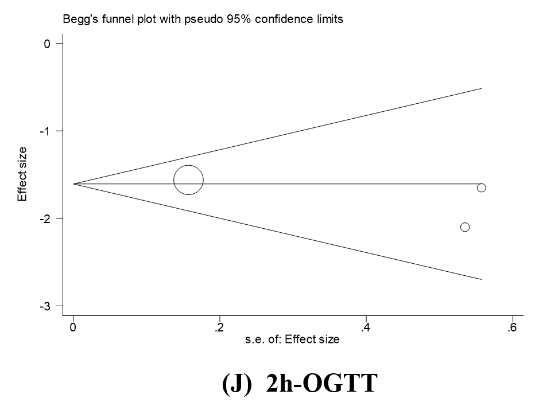


**Figure S3.7.** Begg's test for assessing publication bias. (A) TG Begg's test (*p* = 0.048); (B) HDL-C Begg's test (*p* = 0.754); (C) LDL-C Begg's test (*p* = 0.386); (D) BMI Begg's test (*p* = 0.711); (E) TC Begg's test (*p* = 0.548); (F) FPG Begg's test (*p* = 0.707); (G) WC Begg's test (*p* =1.000); (H) SBP Begg's test (*p* = 0.296); (I) DBP Begg's test (*p* = 1.000); (J) 2h OGTT Begg's test (*p* = 1.000).


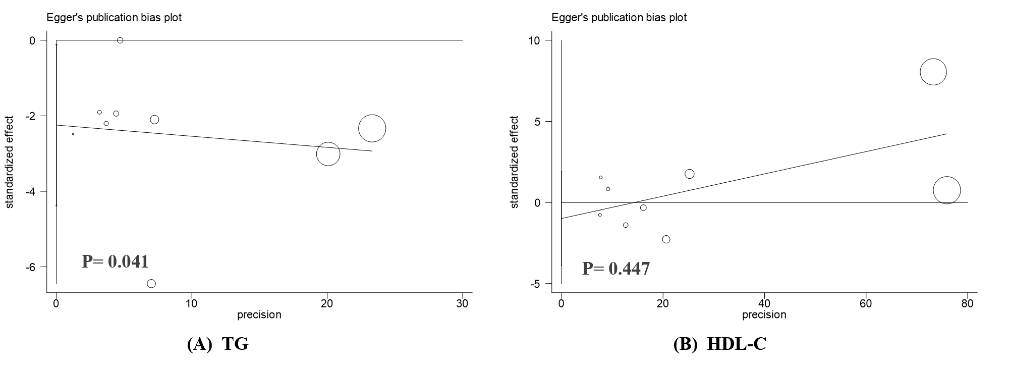

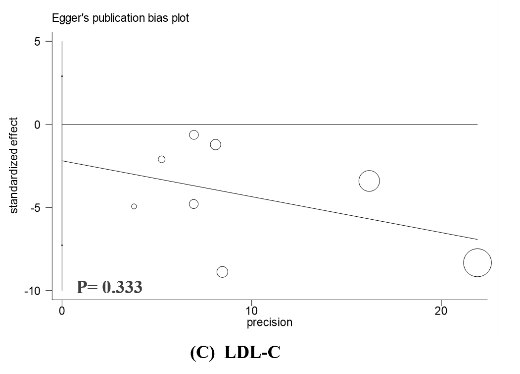


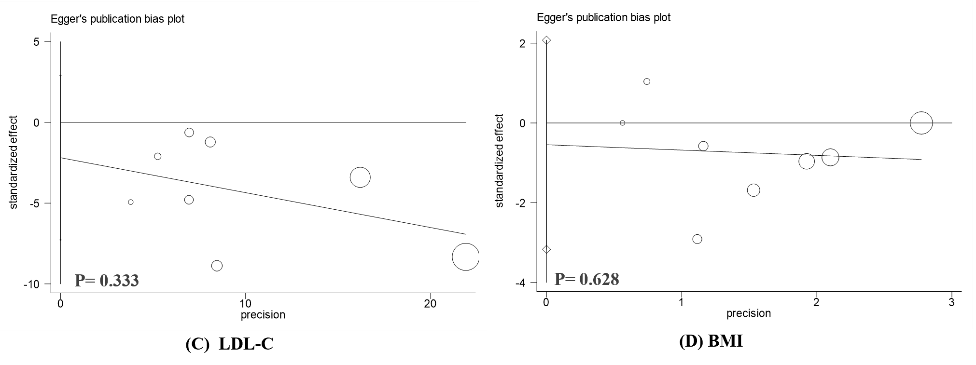

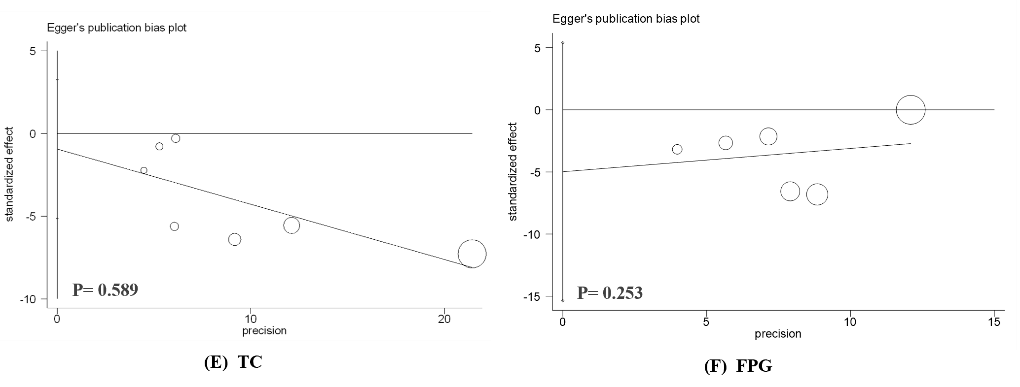


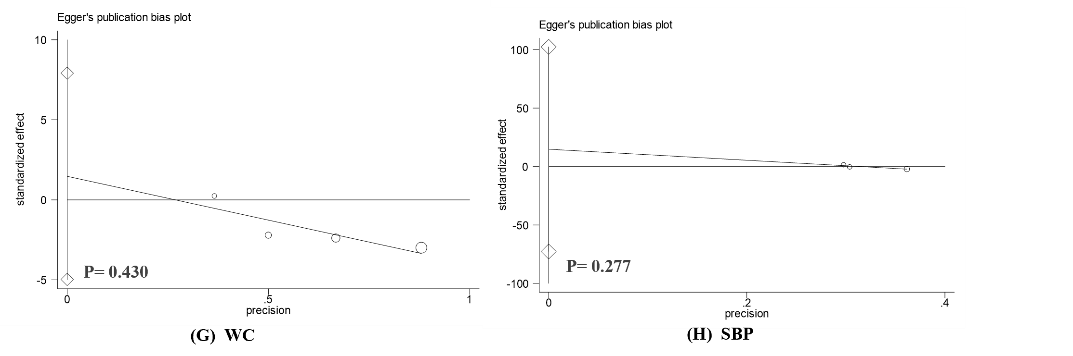

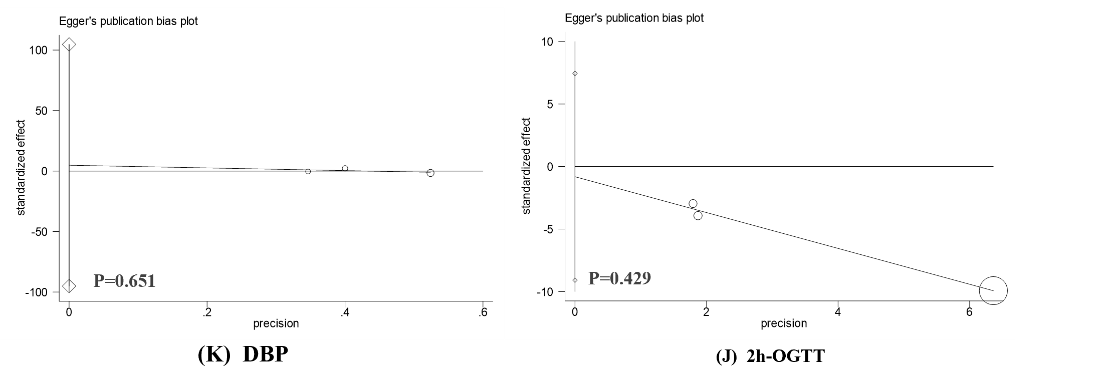


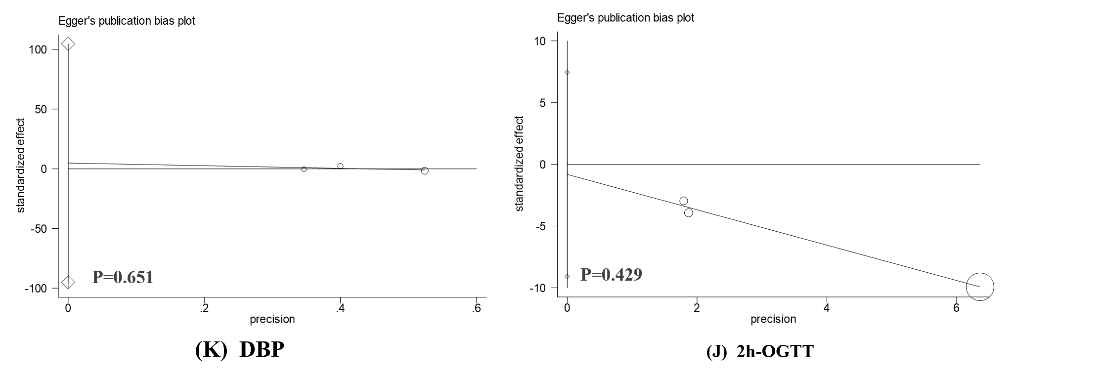


**Figure S3.8.** Egger's test for assessing publication bias. (A) TG Egger's test(*p* = 0.041); (B) HDL-C(*p* = 0.447); (C) LDL-C(*p* = 0.333); (D) BM(*p* = 0.628)I; (E) TC(*p* = 0.589); (F) FPG(*p* = 0.253); (G) WC(*p* = 0.430); (H) SBP(*p* = 0.277); (I) DBP(*p* = 0.651); (J) 2h OGTT(*p* = 0.429).

**
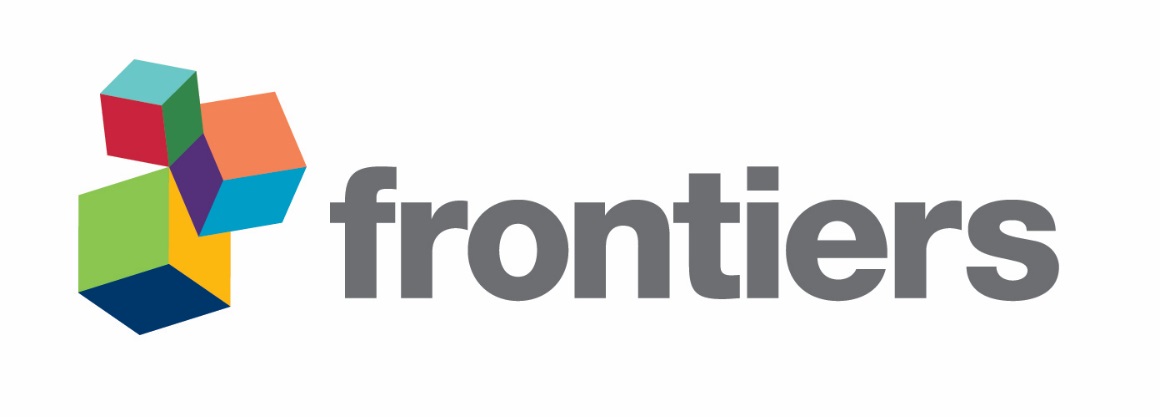
**
